# Chromosome Structuring Limits Genome Plasticity in Escherichia coli

**DOI:** 10.1371/journal.pgen.0030226

**Published:** 2007-12-14

**Authors:** Emilie Esnault, Michèle Valens, Olivier Espéli, Frédéric Boccard

**Affiliations:** Centre de Génétique Moléculaire du CNRS, 91198 Gif-sur-Yvette, France; Université Paris V, INSERM U571, France

## Abstract

Chromosome organizations of related bacterial genera are well conserved despite a very long divergence period. We have assessed the forces limiting bacterial genome plasticity in Escherichia coli by measuring the respective effect of altering different parameters, including DNA replication, compositional skew of replichores, coordination of gene expression with DNA replication, replication-associated gene dosage, and chromosome organization into macrodomains. Chromosomes were rearranged by large inversions. Changes in the compositional skew of replichores, in the coordination of gene expression with DNA replication or in the replication-associated gene dosage have only a moderate effect on cell physiology because large rearrangements inverting the orientation of several hundred genes inside a replichore are only slightly detrimental. By contrast, changing the balance between the two replication arms has a more drastic effect, and the recombinational rescue of replication forks is required for cell viability when one of the chromosome arms is less than half than the other one. Macrodomain organization also appears to be a major factor restricting chromosome plasticity, and two types of inverted configurations severely affect the cell cycle. First, the disruption of the Ter macrodomain with replication forks merging far from the normal replichore junction provoked chromosome segregation defects. The second major problematic configurations resulted from inversions between Ori and Right macrodomains, which perturb nucleoid distribution and early steps of cytokinesis. Consequences for the control of the bacterial cell cycle and for the evolution of bacterial chromosome configuration are discussed.

## Author Summary

Genomic analyses have revealed that bacterial genomes are dynamic entities that evolve through various processes including intrachromosome genetic rearrangements, gene duplication, and gene loss or acquisition by gene transfer. Nevertheless, comparison of bacterial chromosomes from related genera revealed a conservation of genetic organization. Most bacterial genomes are circular molecules, and DNA replication proceeds bidirectionally from a single origin to an opposite region where replication forks meet. The replication process imprints the bacterial chromosome because initiation and termination at defined loci result in strand biases due to the mutational differences occurring during leading and lagging strands synthesis. We analyze the strength of different parameters that may limit genome plasticity. We show that the preferential positioning of essential genes on the leading strand, the proximity of genes involved in transcription and translation to the origin of replication on the leading strand, and the presence of biased motifs along the replichores operate only as long-term positive selection determinants. By contrast, selection operates to maintain replication arms of similar lengths. Finally, we demonstrate that spatial structuring of the chromosome impedes strongly genome plasticity. Genetic evidence supports the presence of two steps in the cell cycle controlled by the spatial organization of the chromosome.

## Introduction

Genomic analyses have revealed that bacterial genomes are dynamic entities that evolve through various processes, including intrachromosome genetic rearrangements, gene duplication, and gene loss or acquisition by lateral gene transfer [1]. Nevertheless, comparison of bacterial chromosomes from related genera revealed a conservation of organization [2]. For example, the genetic maps of E. coli and Salmonella typhimurium that diverged from a common ancestor about 140 million years ago are extensively superimposable [1]. Multiple forces seem to shape the organization of bacterial chromosomes, and the imprinting of these processes on the chromosome is evident at different levels.

DNA replication initiated at *oriC* proceeds bidirectionally until the two replication forks meet. Replication initiation and termination at defined loci result in guanine/cytosine skew between leading and lagging strands due to the mutational differences [3–5]. In wild-type (wt) cells, replication arms coincide with the two compositional skewed halves of the chromosome, hence the name of replichore [6]. Initiation of replication occurs at *oriC*, the origin junction of replichores, and in most cases, the two replication forks are predicted to meet at the terminal junction of replichores where skew changes [7]. Biological processes may exploit these strand-biased sequences defining each replication arm as a target for selection pressure. Two examples of positive selection at the replichore scale have been well documented in bacteria; first, the octamer χ sequence involved in the RecBCD-mediated recombination process is overrepresented 3.5 times in one orientation along each replichore [8]. Second, FtsK-Orientating-Polar-Sequences (KOPS) are overrepresented on one DNA strand ([9], see below).

Beyond the replichore organization, processes affecting the genome organization at the gene level also shape chromosome structures, and two different parameters might be affected: orientation of gene transcription relative to replication, and location of genes relative to the origin of replication. Since replication and transcription occur simultaneously on the same DNA molecule, both head-on and co-oriented collisions are thought to occur in replicating bacteria. It has been originally proposed that highly expressed genes are preferentially positioned on the leading strand to allow faster DNA replication and reduce transcript losses that occur during head-on collisions [10]. In E. coli, 54% of coding sequences are found on the leading strand, and as for most bacterial species, highly expressed genes such as rRNA operons (rDNA) and genes encoding ribosomal proteins are transcribed in the direction of replication. However, at least in E. coli and Bacillus subtilis, essentiality, not expressiveness, selectively drives the gene-strand bias [11]. Another parameter thought to shape chromosome structure at the gene level involves the location of genes relative to the replication origin, and gene dosage effect may constrain this positioning. In fast-growing bacteria, the replication gene dosage effects are mainly associated with the elements of the translation and transcription machinery, i.e., rDNA, transfer DNA (tDNA), RNA polymerase, and ribosomal protein genes [12].

**Figure 1:**
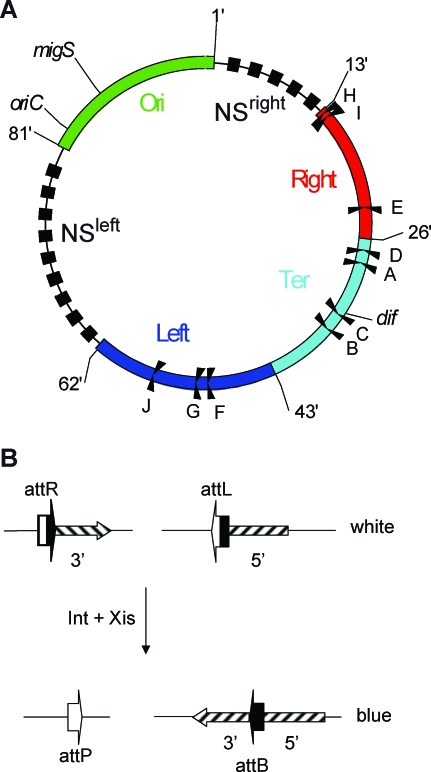
Macrodomain Organization of the E. coli Chromosome (A) Schematic representation of E. coli MDs and nonstructured (NS) regions. The circle represents the genetic map of the chromosome. Colored bars represent the different MDs (with their coordinates indicated in minutes), and interrupted black bars schematize the two NS regions. The ten Ter sites (from A to J), replication origin *oriC*, *migS*, and *dif* sites are indicated. (B) Integrative and excisive recombination promoted by Int + Xis. In the presence of Int and Xis proteins, recombination between *att*L and *att*R sites generates *att*B and *att*P sites. It recreates a *lacZ* coding sequence allowing phenotypic detection of recombined fragments.

In bacteria, selection operates to maintain the two replichores of approximately equal length. In most cases, the size of the longest replichore corresponds to 50%–60% of the entire chromosome [13]. In E. coli, the constraint on the size of replication arms is ensured by the presence of ten Ter sites (TerA–J) scattered in two oppositely oriented groups in the terminal half of the chromosome ([14], Figure 1A). Each of the Ter sites binds Tus, the replication terminator protein, with a specific affinity. Each replication fork travels across the five Ter sites in the permissive orientation before it encounters a Ter site in the nonpermissive orientation and is blocked. The forks are thus trapped between oppositely oriented sites, defining a region called the replication fork trap. In conditions in which Tus blocks replication forks at ectopic Ter sites, creating a region impossible to replicate, the RecBCD pathway of homologous recombination and SOS induction are essential for viability [15–17]. The need for a high level of homologous recombination protein RecA and helicase UvrD accounts for the requirement of SOS induction for viability [18]. A detailed study has shown that forks blocked at Ter sites are stable; linear DNA molecules are formed upon arrival of a second round of replication forks and RecBCD-promoted recombination catalyzes the reincorporation of the double-strand DNA (ds-DNA) ends made by replication run off [17]. UvrD was proposed to enable replication forks initiated at recombination intermediates to progress across the Ter–Tus barrier [18].

Microscopy observations have shown that circular bacterial chromosomes are organized with a particular orientation within growing cells that preserves the linear order of loci on the DNA [19–23]. The E. coli chromosome consists of four structured macrodomains (MDs) and two nonstructured regions [24,25]. The Ori MD containing the origin of replication *oriC* is centered on *migS*, a centromere-like structure involved in bipolar positioning of *oriC* [26]. The Ori MD is flanked by two nonstructured (NS) regions called NS^right^ and NS^left^ (Figure 1A). The Ter MD containing the replication fork trap is centered on the terminal replichore junction. The Ter MD is flanked by two MDs called the Right and Left MDs. The existence of the four MDs and two NS regions was deduced from genetic data showing that different MDs do not interact during cell growth, but interact with their adjacent NS regions [25]. Several important processes take place in the Ter MD. First, replication ends in the Ter MD because of the presence of the replication fork trap. Second, the replichore junction diametrically opposed to *oriC* is the region of the change in compositional skew defining the two replichores [7]. The site-specific recombination site *dif* is present near the replichore junction and allows the resolution of chromosome dimers into monomers; to be active, *dif* must be present in a zone of converging KOPS [9,27]. KOPS are recognized by FtsK which translocates the DNA directionally in order to align *dif* sites at the septum where XerCD can resolve chromosome dimers into monomers (for review, see [28,29]). Third, the Ter MD contains two Non-Divisible Zones (NDZ) refractory to inversions ([30], see below).

Genetic approaches have provided experimental evidence that some chromosome rearrangements are detrimental for growth or, in rare cases, refractory to inversions [30–37]. Using homologous recombination, intrareplichore inversions (Intra) of segments with one endpoint located in the 20%–30% region flanking the terminal replichore junction, i.e., the periphery of the Ter MD, have been shown to be reproducibly highly problematic or prohibited in E. coli (for review, see [38]). However, these regions are not refractory to inversions by the site-specific recombination system used here [25,37]. Inversions that split the Ter MD are detrimental for growth and delay cell division [37].

In a previous study, we have generated strains with chromosomes carrying inverted segments using the λ site-specific recombination system [25]. Interestingly, we noticed that strains carrying combinations of partner *att* sites located in the same regions of the chromosome have similar phenotypes upon inversion, and many of the inversions seemed to affect cell physiology. The results reported here allow us to define extents and limits to plasticity in the E. coli chromosome. The analysis of detrimental rearrangements allowed the identification of two types of chromosome inversions that, by changing MD organization, severely affect the progression of the cell cycle.

## Results/Discussion

### A Genetic System to Change Chromosome Configuration

By using the site-specific recombination system of bacteriophage λ, we previously developed a genetic system that allows the construction and detection of genetic inversions in the E. coli chromosome [25]. We have constructed several series of strains containing one defined *att* site at a fixed position and its *att* partner site inserted at random locations; strains carrying combinations of partner *att* sites that could give rise to viable recombinants have been selected. Cassettes were designed to detect inversion between *att* sites: recombination between *att*L and *att*R restores *lacZ* integrity (Figure 1B). By providing a limiting amount of recombinase, we were able to reveal the existence of MDs that correspond to large regions that are insulated from each other in the cell (Figure 1A). By providing a high amount of recombinase, recombination between most of the combinations of *att* sites can be detected, and there is a good correlation between the frequency of collisions and the frequency of recombinants [25]. There was no correlation between the frequency of inversion and the physiological properties of cells with inverted configuration; inversions occurring at high frequency can be detrimental, whereas those occurring at low frequency can be neutral (see below). We now analyze in detail the properties of strains carrying chromosomes with the different inverted configurations.

To unravel the consequences of inverting a chromosomal segment on cell physiology, we performed a number of analyses aimed at detecting defects visible at the colony or cell level. The size of colonies from strains with the inverted chromosome (*lacZ* reconstituted, blue colonies) was compared to that of strains with the wt configuration (white colonies) in rich medium. The effect of these inversions on growth was also measured using a coculture assay in which strains with chromosomes in wt and inverted configurations were compared (Materials and Methods). To analyze the consequences of the inversion on the nucleoid morphology, cells grown in exponential phase were stained with DAPI, and nucleoids were observed by fluorescence microscopy (see Materials and Methods). The percentages of cells with different types of nucleoids were numbered according to the cell size. The number of chromosome origins was estimated by fluorescence-activated cell sorting (FACS) analysis. Viability of strains was tested in different genetic backgrounds affected in pathways related to DNA metabolism. We used *recA* mutants and since RecA is required for both homologous recombination and SOS induction, the requirement of SOS induction for viability was tested in *lexA ind^−^* (SOS^−^ Rec^+^) and *recA lexA^def^* (SOS^+^ Rec^−^) mutants. When RecA was required, we used mutants affected in the two RecA-dependent recombination pathways, i.e., RecBC and RecFOR, to identify the pathway involved. Measurements of SOS induction were performed in a *sfiA* background to avoid SfiA-dependent filamentation and inhibition of cell division [39]. SOS induction was quantified in culture by using a plasmid carrying the *uidA* gene encoding β-glucoronidase under the control of the P_sfiA_ promoter (see Materials and Methods). In addition, the presence of a plasmid carrying a *gfp* gene under the control of the P_SfiA_ promoter allowed the direct visualization of the induction of the SOS response at the cellular level. *tus* mutants were used to estimate the defects provoked by inverted Ter sites in various configurations. When recombinant colonies could not be obtained, PCR reactions probing the presence of recombination at the DNA level were used to check for the occurrence of *att*L–*att*R inversion and for the presumed lethality conferred by the inversion.

### Ter Sites Impede Replication Forks with Various Efficiencies

Ten Ter sites are found in the E. coli chromosome, which are bound in vitro by Tus with varying efficiencies [14]. The *K*_obs_ for Tus binding to the very strong TerB site is about 5 × 10^−13^ M, and the relative arrest activity was estimated to be around 95%. Although studies were not performed with other Ter sites, the effect of mutations in TerB mimicking the sequence of other Ter sites allows estimation of their respective strength as deduced from Tus–Ter binding affinity measurements and from measure of the replication arrest activity [14]. TerA–E and TerG are predicted to be very strong sites (arrest activity greater than 50%), TerH moderately strong (arrest activity around 33%), and TerF and TerI–J weak sites (arrest activity less than 20%). To estimate the respective strength of Ter sites in vivo in a strain producing wt levels of Tus, we have generated strains in which different Ter sites are inverted, and their properties have been analyzed (Figures S1 and S2, and Text S1). Altogether, the results indicate that efficiency of replication arrest at different chromosomal Ter sites correlates with the predictions based on in vitro affinities and on replication arrest activity of TerB mutant sites. They show that in conditions of wt level of Tus protein, blocking the two forks by the strong TerE and TerA sites renders RecBCD-dependent recombination essential for viability, as previously observed with TerA [15]. SOS induction is also essential in these conditions. The effect of the moderate site TerH in the inverted orientation was less severe, but still significant. Inverted weak TerI and TerJ sites do not appear to affect growth, suggesting they do not significantly impede replication (Figure S1 and Text S1).

### Imbalance of Replication Arms Renders RecBC Essential for Growth

**Figure 2:**
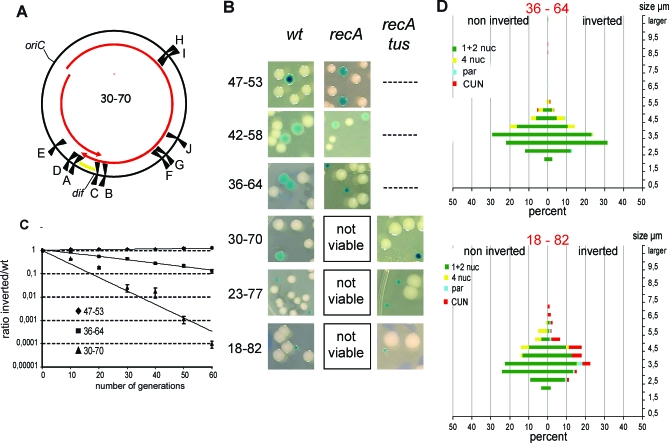
Imbalance of Replication Arms (A) Schematic representation of the E. coli chromosome after interreplichore inversion leading to a 20% imbalance (replication arms are 30% and 70% of the total length of the chromosome). The two replication arms are indicated by red arrows, and the replication fork trap is indicated in yellow. The ten Ter sites (from A to J), replication origin *oriC*, and *dif* site are indicated. (B) Colonies of strains carrying chromosomes with various levels of imbalance as indicated (47–53: strain Inter R-L3; 42–58: strain Inter R-L5; 36–64: strain Inter R-NS^left^1; 30–70: strain Inter R-NS^left^2; 23–77: strain Inter R-NS^left^4; and 18–82: strain Inter R-O1). Colonies were obtained in a wt, *recA*, or *recA tus* genetic background. (C) Quantification of the growth defects caused by replication arms imbalance. Strains carrying a chromosome with 3% (47–53), 14% (36–64), or 20% (30–70) of replication arms imbalance described in (B) were grown in serial cocultures with the strain carrying the wt configuration. The ratio of inverted to wt configurations is plotted as a function of the number of generations. (D) Comparison of microscopic analyses of strains carrying a chromosome with 14% (36–64) or 32% (18–82) of replication arms imbalance described in (B). Colored horizontal bars indicate the percentage of the different types of cells and nucleoids in the wt (noninverted; left) and inverted (right) configurations. Green indicates cells containing one and two nucleoids (1+2 nuc); yellow: cells containing four nucleoids (4 nuc); cyan: cells containing par-like nucleoids (par); red: cells with unsegregated nucleoid (CUN); and pink: anucleate (anuc).

On the E. coli chromosome, the two replichores are of similar size, suggesting that most replication forks meet within the replication fork trap diametrically opposite to the origin. To evaluate the requirements for the balance of replication arms, we analyzed strains in which inversion endpoints are in each replication arm, asymmetrically relative to *oriC* (interreplichore inversion [Inter], Figure 2A, Table 1). As the inverted region contains *oriC*, these inversions do not change the orientation of sequences or genes relative to replication. However, because the two endpoints are at different distances from *oriC*, the size of the replication arms are modified, one becoming greater and one smaller than 50% of the chromosome. Imbalance of 5%–10% for replication arms has no effect on colony morphology: the colonies with inverted configurations are similar to those with wt configuration (Figure 2B, 47% for the short arm and 53% for the long arm (47–53) in strain Inter R-L3 (Table 1), and 42–58 in strain Inter R-L5 (Table 1)). The effect of these inversions on growth was also measured using the coculture assay containing strains with either a chromosome in wt or inverted configuration: no defect was associated to this genetic rearrangement as the ratio of inverted to wt cells was close to one after 60 generations (Figure 2C and unpublished data). The cells and nucleoids of strains with either configuration were not distinguishable (Figure S3). When the imbalance reached 15% (36–64 in Figure 2B, strain Inter R-NS^left^1 in Table 1), some defects became apparent. The recombinant colonies were smaller than noninverted colonies, and the ratio of inverted to wt cells after 60 generations was affected (0.13 ± 0.02), but the cells and nucleoids of the inverted configuration were similar to those of the wt configuration: only 2% of the cells appeared abnormal (Figure 2D). Around 20% of imbalance (30–70 in Figure 2B, strain Inter R-NS^left^2), the size of colonies carrying the inversion was affected; in coculture assays, the ratio of cells with inversion to wt configuration was less than 0.01 (Figure 2C) and microscopic observation showed longer cells with abnormal nucleoids (14% of abnormal cells in Inter R-NS^left^2, Figure S3). Above 20% of imbalance (23–77 and 18–82 in Figure 2B, strains Inter R-NS^left^4 and Inter R-O1, respectively, in Table 1), colonies were barely visible, and more than 20% of cells displayed condensed nucleoids, i.e., a par phenotype, or grew as cells with unsegregated nucleoids (Figure 2D and Figure S3, respectively).

**Table 1:**
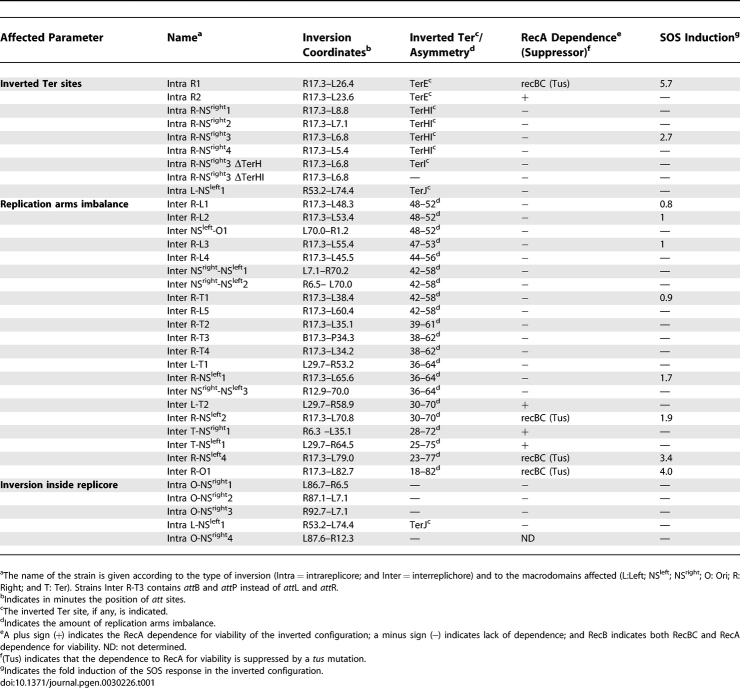
Strains with Rearrangements Generating Inverted Ter Sites, Imposing Imbalance of Replication Arms, and Affecting Gene Orientation

Interestingly, we noticed that all strains with an imbalance greater than 20%, i.e., with a replication arm smaller than 30% and the other larger than 70%, were dependent on RecA for viability (Figure 2B and Table 1). Recombinant colonies could be obtained in a *recFOR* background, but not in conditions inhibiting either RecBC DNA recombination or SOS induction, indicating that the RecBC homologous recombination pathway is required for viability in the presence of an imbalance of replication arms greater than 20% (Table 1). The dependence on RecA for viability was suppressed by a *tus* deletion, indicating that the impediment of replication forks by Tus at Ter sites is responsible for lethality in a *recA* background (Figure 2B). Finally, a 2- to 4-fold SOS induction was apparent in strains that required *recA* for viability (Table 1). The analysis performed with inverted Ter sites indicated that in cells expressing wt levels of Tus, the replication forks are stopped at the first strong Ter site in the nonpermissive orientation ([15] and Text S1). It implies that, when the imbalance is smaller than 20%, the two forks of a same replication round can progress to the replication fork trap. In contrast, RecBC-dependent recombination is solicited to restart the first fork that reaches a Ter site before the other fork can reach it when the imbalance of replication arms is larger than 20%. We propose that, in the conditions used, when the shorter replication arm is less than half the longer one, it is fully replicated twice before completion of replication of the longer arm, leading to the formation of DNA double-stranded ends. These double-stranded ends induce the SOS response and are lethal in the absence of RecARecBC-dependent homologous recombination. Many natural inversions in bacterial genomes are symmetrical with respect to replication origins and termini. Scatter plots of the conserved sequences between related species produce an X-shaped pattern, called X-alignment [2]. These rearrangements reveal that selection operates to maintain replichores of similar lengths; in most genomes, the size of the longest predicted replication arm does not exceed 60% of the chromosome [13]. By moving the position of the replication fork trap on the genetic map, we have been able to analyze the effect of varying the imbalance of replication arms. Remarkably, we did not observe negative effects when the imbalance was around 10%, in total agreement with the observed size distribution of replichores in different species. Some defects appeared when the imbalance reached 15%, and recombinational rescue of replication forks was required above 20%.

The analysis of interreplichore inversions affecting at the same time two MDs revealed that making hybrid MDs while keeping the wt replichore junction unaffected was well tolerated (Figure S3). We noticed that for similar levels of imbalance less than 20%, inversions involving endpoints located either in the NS regions or in the Left, Right, and Ter MDs (Figure S3 and Table 1) behave similarly: the growth of recombinant colonies was slightly affected, and recombinants were viable in a *recA* background (Table 1). It is only when the imbalance exceeded 20% that recombinant colonies were affected and their formation *recA*-dependent (Table 1). Altogether, these results suggest that in the context of interreplichore inversions, the effect of MD disorganization for the Left, Right, and Ter MDs can be well tolerated by the cell.

### Large Intrareplichore Inversions between NS Regions and MD Are Not Detrimental for Growth

**Figure 3:**
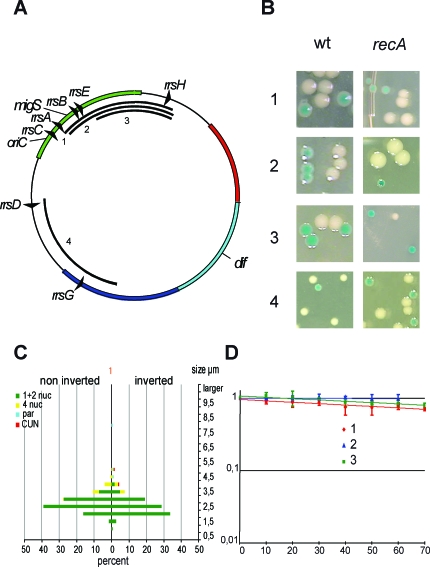
Tolerance to Large Inversions within a Replichore (A) Schematic representation of the E. coli chromosome showing the MDs (Ori in green, Right in red, Left in blue, and Ter in cyan), the seven *rrn* operons (*rrsA* to *rrsH*), replication origin *oriC*, *migS*, and *dif*. The numbered black arcs indicate the position and extent of fragments inverted in the strains 1 to 4 (1: intra O-NS^right^1; 2: intra O-NS^right^2; 3: intra O-NS^right^3; and 4: intra O-NS^left^1). (B) Colonies of strains 1 to 4 carrying normal and inverted configurations. Colonies were obtained in a wt or *recA* genetic background. (C) Comparison of microscopic analyses of cells from strain 1 in wt (noninverted) and inverted configurations as described in Figure 2. (D) Quantification of the growth defects caused by intrareplichore NS^right^–Ori inversions. Strains 1 to 3 carrying the inverted configuration were grown in serial cocultures with the strain carrying the wt configuration. The ratio of inverted to wt configurations is plotted as a function of the number of generations.

We noticed that large inversions inside a replichore (intrareplichore) with one endpoint in the Ori MD and the other in the NS^right^ region gave rise to recombinants with no strong defects. Three examples of strains with such rearrangements are shown in Figure 3. These inversions encompass 916, 927, and 668 kb corresponding to 826, 828, and 607 genes, including four, three, and one rDNA operons, respectively (Figure 3A, strains Intra O-NS^right^1 to −3 in Table 1). Similar outcomes were obtained in the left replichore. For example, the inversion of a 982-kb–long segment that changes the orientation of 942 genes, including two rDNA operons and 34 ribosomal protein genes (strain Intra L-NS^left^1 in Table 1) had no detectable detrimental effects (Figure 3B and unpublished data). The colonies of strains with the rearranged chromosome had the same size as those with the wt configuration (Figure 3B). The diagram shown in Figure 3C indicates that even the largest inversion has no detectable effect on nucleoid morphology. No strong defect was associated with these genetic rearrangements, because the ratio of inverted to wt configuration was above 0.75 after 60 generations in coculture assays (Figure 3D). Finally, colonies carrying these inverted configurations were viable in a *recA* background, indicating the absence of important DNA damage (Figure 3B). Altogether, these results indicate that the direction of replication can be inverted through hundred of genes, including rDNA genes, without important consequences for growth. Furthermore, the results show that inversions between Ori MD and the NS regions are well tolerated. Similar conclusions were obtained from the analyses of intrareplichore inversions between NS regions and the flanking Right or Left MD in the absence of active Ter sites (Figure S4 and Table 1). Therefore, gene orientation, gene dosage, and sequence skews appear to operate only as long-term positive selection determinants. Our results are in agreement with the evidence [11,40] that weakens the proposed influence of replication on gene orientation [41,42]. However, given the large size of bacterial populations, slightly deleterious effects that can be accredited to positioning rDNA and ribosomal protein genes on the lagging strand are most likely sufficient to eliminate such configurations from the population in long-term evolution.

In contrast to well-tolerated inversions described above, two types of intrareplichore inversions were highly detrimental for the cell: the first type involved endpoints located in the Ter and the Right MDs, and provokes the separation of the replication fork trap from the wt replichore junction. The second type involved inversion between endpoints located in the Ori MD and in the Right MD. Features of these two detrimental configurations are described in detail below.

### Intrareplichore Inversions That Disrupt the Ter Macrodomain Are Deleterious

**Figure 4:**
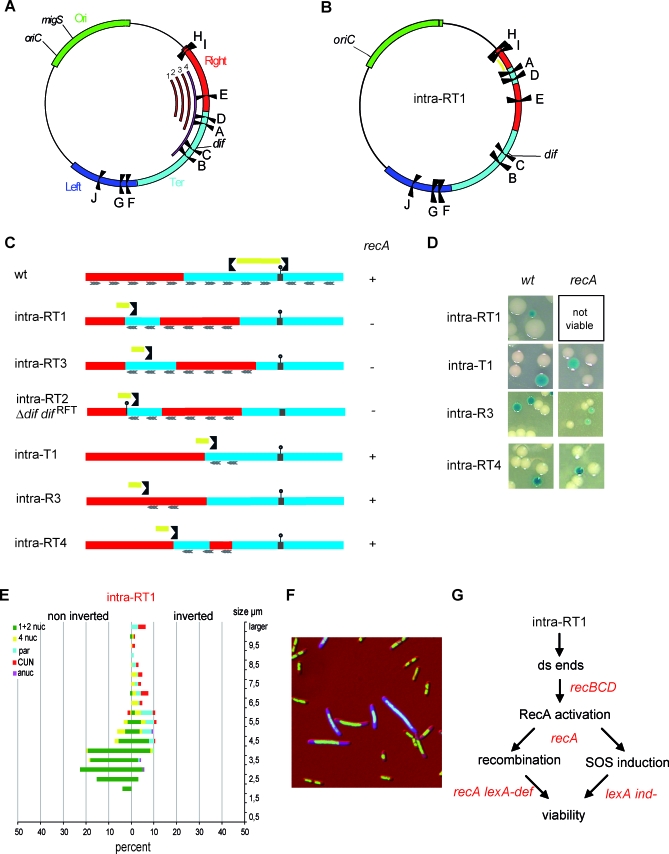
Intrareplichore Inversions between the Right and Ter MDs (A) Schematic representation of the E. coli chromosome showing the MDs (Ori in green, Right in red, Left in blue, and Ter in cyan), the ten Ter sites (from A to J), *oriC*, and *dif*. The numbered red arcs indicate the position and extent of fragments inverted in the strains Intra R-T1 to −3 (1 to 3, respectively), and the pink arc (number 4) shows the fragment inverted in the Inter R-T2 strain. (B) Schematic representation of the E. coli chromosome upon an intrareplichore inversion that causes the intermingling of Right and Ter MDs (strain Intra R-T1). The MDs are colored as in Figure 1, and the replication fork trap is indicated in yellow. The different Ter sites, *oriC*, and *dif* are indicated. (C) Linear genetic maps of the Right and Ter MDs upon various intrareplichore inversions. The inverted Ter site defining the displaced replication fork trap is shown, and the replication fork trap is indicated in yellow. The *dif* site is indicated by a small stick, and the wt replichore junction by a grey box. Inverted KOPS are indicated by rafters (1 for 100 kb). Viability in a *recA* background is indicated by a plus sign (+) beside the map. (D) Colonies of strains carrying chromosomes with various intrareplichore inversions as indicated in (C). Colonies were obtained in a wt or *recA* genetic background. (E) Microscopic analysis of strain Intra R-T1 as described in Figure 2. (F) Cells from strain Intra R-T1 with an inverted configuration were transformed with a plasmid expressing *gfp* under the P_sfiA_ promoter. Nucleoids of cells expressing SOS appear as blue (see Figure S3 for unmerged pictures). (G) Pathways required for viability of the inverted configuration. Genetic backgrounds in which inverted configuration did not give rise to viable colonies are indicated in red.

Intrareplichore inversions with endpoints in the Right and Ter MDs (Figure 4A, strains Intra R-T1 to −3 in Table 2) generate a hybrid Right-Ter MD in which the orientation of TerA, TerD, and TerE is modified, creating a replication arms imbalance close to 35%–65% (see intra R-T1 in Figure 4B). These strains carry two zones of converging KOPS (Figure 4C): the normal one corresponding to the wt replichore junction, and a new one associated with the replication fork trap in the hybrid Right-Ter MD. Inversion severely affected the growth of colonies (Figure 4D). The observation of cells with the inverted configuration revealed the occurrence of a high proportion of abnormal cells: 27% of cells showed a par phenotype, 15% formed cells with unsegregated DNA, and 1% of cells were anucleate (strain Intra R-T1 in Figures 4E, 4F, and S5). Cells larger than 10 μm with a high amount of nonsegregated nucleoids were observed. FACS analyses indicated that the number of chromosomes in the large cells ranged from 16 to 32 (unpublished data). Other strains with intrareplichore inversions between Right and Ter MDs (Intra R-T2 and −3 in Figure 4 and Table 2) showed the same features (unpublished data).

**Table 2:**
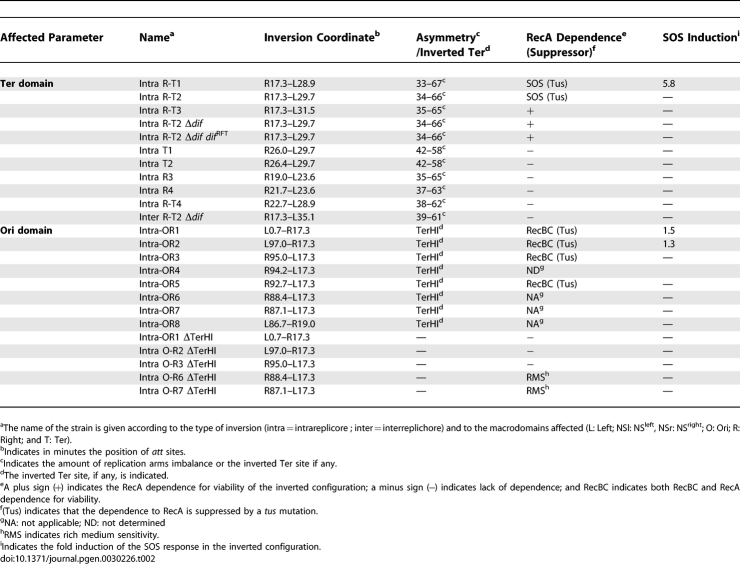
Strains with Rearrangements Making Hybrid Right–Ter MDs and Ori–Right MDs

The origin of the detrimental phenotypes caused by this chromosomal configuration was analyzed by testing different genetic backgrounds (Figure 4G). It was not possible to obtain viable recombinants in a *lexA ind* ^−^ background, i.e., in SOS-defective conditions. SOS induction was directly visualized by the use of a plasmid expressing *gfp* under the control of P_SfiA_ promoter (Figures 4F and S5). Homologous recombination was also required because recombinants with the inverted configuration could not be obtained in a *recA, recBC*, or in a *recA lexA^def^* background (i.e., in conditions of constitutive SOS induction, but in the absence of RecA-dependent recombination).

The phenotype and RecA-independence of interreplichore Right-Ter inversions (Figure S3, strains Inter R-T1 to −4 in Table 1) suggests that intermingling Right and Ter MDs cannot by itself be responsible for the growth defects of strains Intra R-T1 to −3 in the inverted configuration. Growth defects and RecA dependence for viability were suppressed in a *tus* background, indicating that the position of the displaced replication fork trap is responsible for the growth defects (Figure S5E). The detrimental effects can not originate only from imbalance of replication arms because the imbalance of replication arms is close to 35–65, a level that does not render RecA essential for viability in interreplichore inversions (Figure 2 and Table 1). Three other hypotheses that might account for the growth defects were tested below: the positioning of *dif* outside of the replication fork trap, the presence of two zones of converging KOPS, and the merging of replication forks far away from the wt replichore junction region.

In these intrareplichore Right-Ter inversions, the replication fork trap is separated from *dif*. It was previously reported that the *dif* site does not need to be present in the replication fork trap to be fully active because the insertion of a ectopic TerA* site near TerA, moving the replication fork trap away from the *dif* region, did not affect *dif* activity [43]. *dif* is active in any new replichore junction formed after inversion [9,27]. After deletion of *dif* from its normal position, we reinserted a 28-bp fragment corresponding to *dif* in the new replication fork trap, in the region where KOPS converge, far away from the wt replichore junction (Figure 4C, strain Intra R-T2 Δ*dif dif^RFT^* in Table 2). Strains carrying this inverted configuration still showed strong detrimental defects and were not obtained in a *recA* background (Table 2) even though insertion of *dif* in the new replication fork trap improved nucleoid distribution in a way suggesting *dif* activity, i.e., by removing a 12%–15% fraction of filaments (47% of abnormal cells instead of 64% in the absence of *dif,* and 50% when *dif* is present at its normal location; unpublished data). These results indicate that the absence of *dif* from the new replication fork trap is not responsible for the observed defects. These results were corroborated by the viability of Inter Right-Ter inversions in a *recA* background when *dif* was deleted (strain Inter R-T2 Δ*dif* in Table 2), confirming that the RecA dependence for viability of Intra Right-Ter inversions does not result from the lack of *dif* in the replication fork trap.

To analyze the defects provoked by forming two zones of converging KOPS and by positioning the replication fork trap far from the *dif* region but without Right and Ter MDs intermingling, we constructed strains with an inversion that positioned the replication fork trap at the limit between the Ter and the Right MDs (Intra T1 in Figure 4C, and strains Intra T1 and Intra T2 in Table 2). Colony formation was not affected; cells and nucleoids were similar to those of the wt configuration, and strains carrying inversions were viable in a *recA* background (Figure 4C and 4D). These results indicate that as long as sequences belonging to the Ter MD remain together, merging of replication forks far away from the wt replichore junction in the presence of two zones of converging KOPS does not provoke important growth defects.

To determine whether merging of replication forks outside the Ter MD may be responsible for the detrimental effects of intrareplichore Right-Ter inversions, we generated two different genetic inversions in the Right MD (strain Intra R3 in Figure 4C, and strains Intra R3 and Intra R4 in Table 2) that inverted TerE in a strain in which TerA and TerD are deleted; inversion of the TerE region provoked replication to end in the Right MD, and generated two zones of converging KOPS (Figure 4C) and an imbalance of replication arms close to 35–65 (Table 2). Recombinant colonies were slightly affected compared to those with a wt configuration (Figure 4D); cells and nucleoids from both configurations were similar (unpublished data), and recombinants were viable in a *recA* background (Figure 4D). These results indicate that replication forks can merge in the Right MD without affecting viability.

Because none of the simple modifications in the chromosome structure can, by itself, account for the growth defect of intrareplichore Right-Ter inversions, we tested whether the defect was dependent on the length of the Right MD that separates the replication fork trap from the wt replichore junction in the Intra R-T1 configuration. We constructed strain Intra R-T4 (Figure 4C and Table 2). In this strain, the chromosome configuration is similar to Intra R-T1, Intra R-T2 and Intra R-T3 configurations, but the extent of sequences belonging to the Right MD that are embedded in the Ter MD is reduced (170 kb compared to 420 kb). Recombinants showed fewer defects; only a fraction of cells (13%) showed a par phenotype, and less than 1% formed cells with unsegregated nucleoids (Figure S6). Importantly, strains in the inverted configuration were viable in a *recA* background (Figure 4D). These results are in agreement with the hypothesis that the extent of Right MD DNA that separates the Ter region where fork merge from the replichore junction region is responsible for the observed defects. The combination of the embedding of Ter sequences in the Right MD and finishing replication within these Ter sequences is responsible for the deleterious effect. The shortening of the region of Right MD that separate the replication fork trap from the wt replichore junction region suppresses the defects. Together with the observed viability of *recA-* interreplichore inversions involving the Right and the Ter MDs (strains Inter R-T1 and R-T2 in Figure S3 and Table 1), these observations support the hypothesis that the requirement of RecA for the viability of intrareplichore Right-Ter inversions results from the separation of the replichore junction region from the region in the Ter MD where replication ends. It is therefore tempting to speculate that in deleterious configurations resulting from intrareplichore inversion, replication ends in the displaced part of the Ter MD, activities normally associated to the wt replichore junction region cannot be performed, and the cell cycle is affected. Altogether, these results suggest the existence of a tight temporal and/or spatial coupling between the end of DNA replication in the Ter MD and an unknown activity near the replichore junction region required to progress in the cell cycle. Further work will be required to determine whether proteins known to function near the terminal replichore junction, FtsK [44] and TopoIV [45], are involved in this coupling.

### Intrareplichore Inversion That Intermingle Ori and Right MDs Are Detrimental

**Figure 5:**
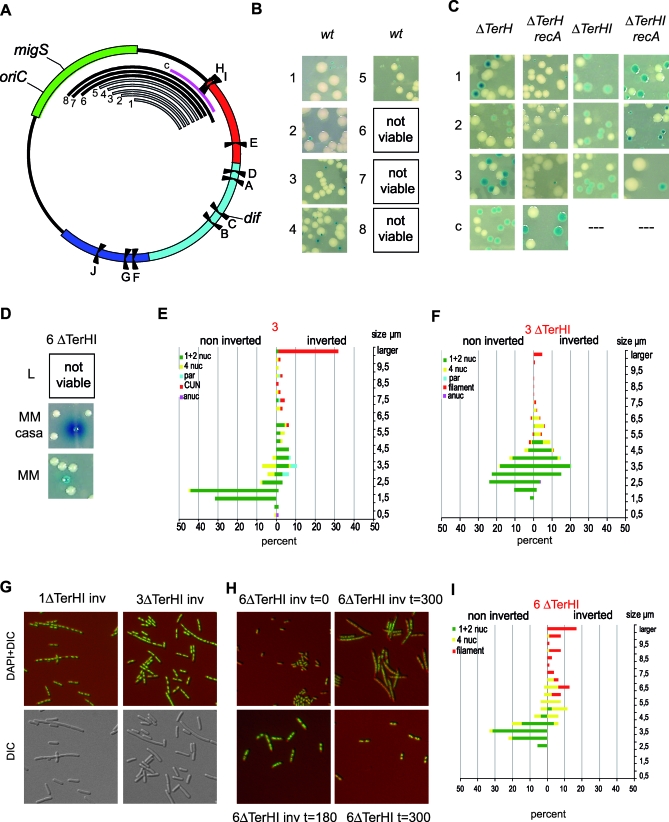
Intrareplichore Inversions between the Right and Ori MDs (A) Schematic representation of the E. coli chromosome showing MDs, Ter sites, *oriC*, *migS*, and *dif*. The numbered black arcs indicate the position and extent of fragments inverted in strains 1 to 8 and (1 to 8: intra O-R1 to −8 in Table 2), and the control strain c is indicated in pink (control Intra R-NS^right^3 in Table 1). (B) Colonies of strains carrying chromosomes with various intrareplichore inversions as indicated in (B). Inversion tests for strains 1 to 8 were performed in rich medium. Colonies carrying the inverted configuration of strains 1 to 5 can be obtained in rich medium (indicated by a grey arc), whereas colonies carrying the inverted configuration of strains 6 to 8 cannot be obtained (indicated by a black arc). (C) Colonies of strains 1 to 3, in the absence of TerH or TerH and TerI, carrying the chromosome in the wt or inverted configurations. Colonies were obtained in a wt or in a *recA* background. Also shown are the results obtained after deletion of the TerH site from strain c in a wt or *recA* genetic background. (D) Colonies of strain 6 deleted for TerH and TerI, in the wt or inverted configuration, obtained in minimal medium (MM) or minimal medium supplemented with casamino-acids (MM casa). (E) Microscopic analysis of strain 3 carrying a chromosome in the wt (noninverted) and inverted configurations as described in Figure 2. (F) Nucleoid and cell analysis obtained from strain 3 deleted for TerH and TerI, carrying a chromosome in the wt or inverted configuration. The left and right colored horizontal bars indicate the percentage of the different types of cells and nucleoids in the wt and inverted configurations, respectively (green indicates cells containing one and two nucleoids; yellow: cells containing four nucleoids; cyan: cells containing par-like nucleoids; red: filamentous cells with apparently segregated nucleoids; and pink: anucleate cells). (G) Images of combined DIC-DAPI and DIC-stained cells from strains 1 and 3 deleted for TerH and TerI, in the inverted configurations, showing the presence of filamentous cells with segregated nucleoids. (H) Combined images of DIC and DAPI pictures of cells from strain 6 deleted for TerH and TerI, in the inverted (inv) or wt configuration after growth in rich medium for 0, 180, or 300 min. (I) Nucleoid and cell analysis obtained from strain 3 deleted for TerH and TerI, carrying a chromosome in the wt or inverted configuration, after 300 min of growth in rich medium. The left and right colored horizontal bars indicate the percentage of the different types of cells and nucleoids in the wt and inverted configurations, respectively (green indicates cells containing one and two nucleoids; yellow: cells containing four nucleoids; and red: filamentous cells with un-segregated nucleoids).

The second class of detrimental inversions corresponds to intrareplichore inversions that combine Ori MD with the Left or Right MD. Because most of the inversions between the Left and Ori MDs also induce a high imbalance of replication arms, we focused our study on the inversion between Ori and Right MDs. The detrimental effects of intermingling Ori and Right MDs were revealed by combining *att*L and *att*R sites inserted at various positions in the Right (14′, 17′, 19′, and 22′) and Ori (0.7′, 97′, 95′, 94′, 92′, 88′, 87′, and 86′) MDs (Figure 5A, Table 2, and unpublished data). Viable recombinants could be obtained only when the inversion involved sites located between 92′ and 0.7′ in the Ori MD, and they all showed strong growth defects (proximal Ori–Right combinations, strains Intra O-R1 to −5 in Figure 5A [indicated in grey] and 5B). In contrast, viable recombinants could not be obtained when the inverted fragment extended from the Right MD to 88′, 87′, or 86′ (distal Ori-Right combinations, strains Intra O-R6 to −8 indicated in black in Figure 5A). Proximal inversions invert the TerHI sites as inversions between Right MD and NS^right^ region (control c in Figure 5A, Intra R-NS^right^3 in Table 1), whereas distal inversions invert both TerHI sites and the centromere-like sequence *migS* found at 89′. *migS* did not seem to be responsible for the difference observed between the two types of combinations since distal combinations did not give rise to viable recombinants in the absence of *migS* (unpublished data). In the absence of both TerH and TerI (ΔTerHI in Figure 5C), viable recombinant colonies with no strong growth defects could be obtained for proximal inversions, and they are viable in a *recA* background (strains Intra O-R1 to −3 in Figure 5C and Text S1). Distal inversions remained lethal on rich medium when both TerH and TerI were deleted (Figure 5D and unpublished data, and strains Intra O-R6 to −8 in Table 2), but viable colonies could be obtained on minimal growth medium (Figure 5D). These recombinants showed growth defects in minimal medium supplemented with casamino-acids (Figure 5D) and could not be propagated in rich medium (unpublished data).

The proximal Ori–Right inversions that gave rise to viable colonies were used for microscopy analysis (strains Intra O-R1 to −3 in Table 2). In the presence of TerH and TerI, we observed a predominant filamentation with DNA accumulating in nonsegregated nucleoids (e.g., 39% of filaments and 10% of par-like cells in the inverted configuration of Intra O-R3 in Figure 5E, and unpublished data). Analysis of the nucleoids of recombinant colonies deleted for TerH and TerI revealed a high percentage of normal cells. However, in the inverted configurations, a significant proportion of cells formed filaments (5%, 9%, and 22%, according to the strain, Figure 5F and 5G, and unpublished data). Remarkably, these filaments were different from those observed in all other rearrangements described in this study; they showed apparently segregated nucleoids with no division septa between DNA bodies (Figure 5G).

To visualize the defects responsible for the absence of viability in distal Ori–Right combinations in rich medium, cells obtained in minimal medium were grown in liquid rich medium and observed at different time points. After 180 min, cells with inverted configurations accumulated a fraction of abnormal cells (16% of filaments not observed in wt cells; Figure 5H and unpublished data), whereas after 300 min, most of the cells were elongated, with improperly compacted and segregated nucleoids (Figure 5H and 5I). Altogether, these results indicate that intermingling Right and Ori MDs interferes with nucleoid management and formation of the division septum. For both proximal and distal combinations involving Ori–Right MDs, the presence of longer cells with an increased number of segregated nucleoids indicates an inhibition in the formation of a septum of division. It is striking to note that inversions that move the Ori MD close to the Right MD (less than 50 kb from the Right MD; strain Intra O-NS^right^4 in Table 1, Figure S7) slightly affect cell physiology. It is therefore likely that the origin of the defect results from an antagonism between Ori and Right MDs rather than from simply moving Ori sequences on the genetic map. The observation that distal inversions were more problematic than proximal ones suggests that the deleterious effects are proportional to the length of the MD. In B. subtilis, the chromosome partitioning and sporulation protein Spo0J binds eight *parS* sites scattered in the 800-kb region flanking *oriC* [46]. Our results suggest the presence of similar specific sequences in the Ori MD. Imbedding of such putative sequences in the Right MD could be the reason for growth defects. In this regard, it is interesting to note that the NS^right^ region separates Ori and Right MDs and could play a buffer role. We would like to speculate that mixing Ori and Right MD sequences would perturb proper segregation of Ori and Right MD, a step necessary to establish septum division. Further experiments will be necessary to determine whether the perturbation of the spatial control of cytokinesis affected by this type of inversion involves SfiA [39], MinCDE [47], SlmA [48], or other unidentified proteins.

**Figure 6:**
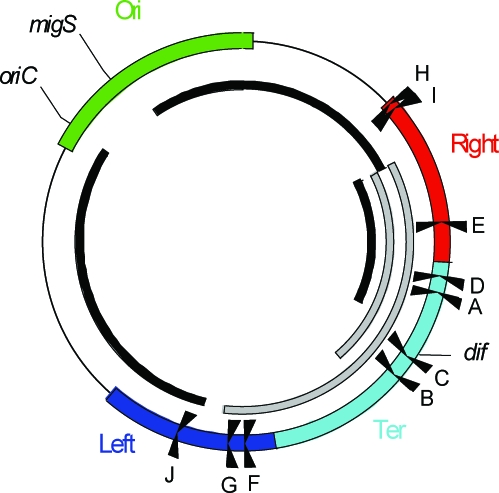
Detrimental Inversions in E. coli Schematic representation of the E. coli chromosome showing MDs, Ter sites, *oriC*, *migS*, and *dif*. The black arcs indicate the position and extent of fragments for which inversion is detrimental for growth. Grey arcs indicate inversions between Right and Ter or Right and Left MDs that are tolerated. Well-tolerated intrareplichore inversions between NS^right^ and adjacent MDs are not indicated. The analysis of detrimental and nondetrimental inversions supports a model in which the Ter MD is involved in a process that couples the end of replication in the Ter MD with a subsequent step. Inversion between Ori and Right MDs results in the perturbation of a process controlling chromosome segregation and cytokinesis.

Altogether, the results reported here give an important insight into the role of MDs in cell cycle control by chromosome configuration in E. coli (Figure 6). The Ter MD is involved in a process that spatially and/or temporally couples the end of replication in the Ter MD with a subsequent step near the replichore junction region. The antagonistic Ori and Right MDs are involved in a process coupling chromosome segregation and cytokinesis. Identification of determinants or factors that specify MDs should help us understand how MDs are involved in the control of these processes.

## Materials and Methods

### Bacterial strains.

E. coli K12 strains are all derivatives of MG1655. Standard transformation and transduction procedures used were as described before [25]. Plasmids and strains with relevant genotypes are described in Table S1.

### Induction.

Conditions for inversion formation were as described previously [25].

### Detection and measurement of the SOS response.

SOS response was quantified by measuring the amount of β-glucoronidase [49] in *sfiA* cells transformed by a pBAD18-derived plasmid carrying the *uidA* gene fused to the *sfiA* promoter. Similar results were obtained in a *sfiA* ^+^ background, but results were less variable in a *sfiA* background. SOS induction was visualized by using cells transformed by a P15A derivative carrying *gfp* under the control of the *sfiA* promoter (pZA-P_sfiA_-*gfp*).

### Microscopy and flow cytometry analyses.

The cultures were grown to optical density (OD) 0.2 at 30 °C and then processed for microscopy or flow cytometry. For the microscopy analysis, the cells were processed as described before [45]. For flow cytometry analysis, chromosome numbering was estimated by counting the number of replication origins using a rifampicin/cephalexin replication run-out [50]; aliquots were taken every 10 min over a period of 200 min. Cells were fixed in a 75% ethanol–PBS 1× solution, then washed in PBS 1×, treated with RNaseA, and the DNA was then stained with propidium iodide. The cells were analyzed on a Partec PASIII flow cytometer.

### Coculture assay.

Strains carrying the inverted configurations were grown in coculture with the same strain carrying the wt configuration. A 1:1 mixture of the two strains was grown in serial cultures in LB medium at 30 °C for up to 70 generations. Every 10 generations, the relative numbers of both configurations were determined by plating. Experiments were performed in triplicate.

## Supporting information

Figure S1 Inversion of Strong and Weak Ter Sites (A) Schematic representation of the E. coli chromosome after the inversion of the strong TerE site. The ten Ter sites (from A to J), the replication origin *oriC*, and *dif* sites are indicated. The two replication arms are indicated by red arrows, and the regions of replication fork blockage by yellow squares.
(B) Colonies of strains carrying TerE, TerHI, TerI, or TerJ in normal (white) or inverted (blue) orientation. Colonies were obtained in a wt, *recA*, or *tus* genetic background, or after deletion of the indicated Ter site (TerE: strain Intra R1; TerHI: strain Intra R-NS^right^3; TerI: strain Intra R-NS^right^3 ΔTerH; and TerJ: strain Intra L-NS^left^1).
(C) Quantification of the growth defects caused by TerHI inversion. Strain Intra R-NS^right^ 3 (Table 1) carrying the inverted TerHI was grown in serial cocultures with the strain carrying the wt configuration. The ratio of inverted to wt cells is plotted as a function of the number of generations. Experiments were performed in wt and *tus* backgrounds (triangles and diamonds, respectively).
(D) Pathway required for viability of the inverted configuration. Genetic backgrounds in which inverted configuration did not give rise to viable colonies are indicated in red.
(E) Comparison of microscopic analyses of cells carrying TerE in a wt and inverted configuration. Colored horizontal bars indicate the size and the percentage of the different cell types and nucleoid contents in the wt (left panel) and inverted configurations (right panel), as described in Figure 2.
(F) Cells with inverted TerE were transformed with a plasmid expressing *gfp* under the P_sfiA_ promoter. Nucleoids of cells expressing SOS appear as blue (see Figure S1 for unmerged pictures).
(1.8 MB AI).

Figure S2 Microscopy Analysis of Cells Carrying TerE in an Inverted Configuration (Strain Intra R1) (A) Phase contrast image of fixed cells.
(B) Fluorescence image of fixed cells expressing *gfp*.
(C) Combined image of phase contrast and fluorescence images of cells fixed and stained with DAPI to reveal nucleoids. Cells are in red and DNA is in green.
(D) Combined image of fluorescence images of cells fixed and stained with DAPI to reveal nucleoids and SOS response. DNA is in green and GFP fluorescence in blue.
(2.7 MB AI).

Figure S3 Imbalance of Replication Arms Nucleoid and cell analyses of strains carrying chromosomes with various levels of imbalance. These inverted configurations were obtained upon interreplichore inversions. For each strain, the genetic map of the chromosome in the inverted configuration is shown. The level of imbalance is indicated above the map, the colonies are shown inside the map. The MDs (Ori in green, Right in red, Left in blue, and Ter in cyan), the ten Ter sites (from A to J), *oriC*, *migS*, and *dif* are indicated. Nucleoid and cell analysis obtained from each strain in both configurations is shown below the map. The left and right colored horizontal bars indicate the percentage of the different types of cells and nucleoids in the wt and inverted configurations, respectively, as described in Figure 2.
(2.5 MB PDF)

Figure S4 Tolerance to Large Inversions within a Replichore Nucleoid and cell analyses of strains carrying intrareplichore inversions between NS^right^ and Ori MD (strain Intra O-NS^right^2), between NS^right^ and Right MD in a strain deleted for TerH and TerI (strain Intra R-NS^right^3 ΔTerHI), and between NS^left^ and Left MD (Intra L-NS^left^1). Nucleoid and cell analysis obtained from each strain in both configurations is shown below the map. The left and right colored horizontal bars indicate the percentage of the different types of cells and nucleoids in the wt and inverted configurations, respectively, as described in Figure 2.
(1.2 MB AI).

Figure S5 Phenotypic Analysis of Strains Carrying a Chromosome with an Intrareplichore Inversion between the Right and Ter MDs (A–D) show cells from strain Intra R-T1 in the inverted configuration; and (E) shows colonies of strain Intra R-T2.
(A) Microscopic phase contrast image of fixed cells.
(B) Microscopic fluorescence image of fixed cells expressing *gfp*.
(C) Combined image of phase contrast and fluorescence images of cells fixed and stained with DAPI to reveal nucleoids. Cells are in red and DNA is in green.
(D) Combined image of fluorescence images of cells fixed and stained with DAPI to reveal nucleoids and SOS response. DNA is in green and GFP fluorescence in blue.
(E) Colonies of strain intra RT2 carrying the wt or inverted configuration in a *tus* or *recA tus* background.
(3.1 MB AI).

Figure S6 Intrareplichore Inversion between the Right and Ter MDs The genetic map of the chromosome in the inverted configuration is shown for strain Intra R-T4 carrying intrareplichore inversion between Ter and Right MDs. The MDs (Ori in green, Right in red, Left in blue, and Ter in cyan), the ten Ter sites (from A to J), *oriC*, *migS*, and *dif* are indicated. Nucleoid and cell analysis obtained is shown below the map. The left and right colored horizontal bars indicate the percentage of the different types of cells and nucleoids in the wt and inverted configurations, respectively, as described in Figure 2.
(1.2 MB AI).

Figure S7 Intrareplichore Inversions between the Right and Ori MDs (A) The genetic map of chromosome in the inverted configuration is shown for strain Intra O-R6 ΔTerHI.
(B) The genetic map of chromosome in the inverted configuration is shown for strain Intra O-R7 ΔTerHI.
(C) The genetic map of chromosome in the inverted configuration is shown for strain Intra O-NSr2. Below the map are shown colonies carrying the wt or inverted configuration. Nucleoid and cell analysis obtained is shown below the map. The left and right colored horizontal bars indicate the percentage of the different types of cells and nucleoids in the wt and inverted configurations, respectively, as described in Figure 2.
(D) The genetic map of chromosome in the inverted configuration is shown for strain Intra O-NSr4. Below the map are shown colonies carrying the wt or inverted configuration. Nucleoid and cell analysis obtained is shown below the map. The left and right colored horizontal bars indicate the percentage of the different types of cells and nucleoids in the wt and inverted configurations, respectively, as described in Figure 2.
(1.4 MB AI).

Table S1 Bacterial Strains and Plasmids (120 KB PDF)

Text S1 Supplementary Text for Chromosome Structuring Limits Genome Plasticity in E. coli
(100 KB PDF)

## Abbreviations

Inter: interreplichore inversion
Intra: intrareplichore inversion
KOPS: FtsK-Orientating-Polar-Sequences
MD: macrodomain
NS: nonstructured
wt: wild type

## References

[pgen-0030226-b001] Lawrence JG, Higgins NP (2005). The dynamic bacterial genome. The bacterial chromosome.

[pgen-0030226-b002] Eisen JA, Heidelberg JF, White O, Salzberg SL (2000). Evidence for symmetric chromosomal inversions around the replication origin in bacteria. Genome Biol.

[pgen-0030226-b003] Lobry JR (1996). Asymmetric substitution patterns in the two DNA strands of bacteria. Mol Biol Evol.

[pgen-0030226-b004] Lobry JR, Louarn JM (2003). Polarisation of prokaryotic chromosomes. Curr Opin Microbiol.

[pgen-0030226-b005] Salzberg SL, Salzberg AJ, Kerlavage AR, Tomb JF (1998). Skewed oligomers and origins of replication. Gene.

[pgen-0030226-b006] Blattner FR, Plunkett G, Bloch CA, Perna NT, Burland V (1997). The complete genome sequence of Escherichia coli K-12. Science.

[pgen-0030226-b007] Hendrickson H, Lawrence JG (2007). Mutational bias suggests that replication termination occurs near the dif site, not at Ter sites. Molecular Microbiology.

[pgen-0030226-b008] El Karoui M, Biaudet V, Schbath S, Gruss A (1999). Characteristics of Chi distribution on different bacterial genomes. Res Microbiol.

[pgen-0030226-b009] Bigot S, Saleh OA, Lesterlin C, Pages C, El Karoui M (2005). KOPS: DNA motifs that control E. coli chromosome segregation by orienting the FtsK translocase. EMBO J.

[pgen-0030226-b010] Brewer BJ (1988). When polymerases collide: replication and the transcriptional organization of the E. coli chromosome. Cell.

[pgen-0030226-b011] Rocha EP, Danchin A (2003). Essentiality, not expressiveness, drives gene-strand bias in bacteria. Nat Genet.

[pgen-0030226-b012] Couturier E, Rocha EP (2006). Replication-associated gene dosage effects shape the genomes of fast-growing bacteria but only for transcription and translation genes. Mol Microbiol.

[pgen-0030226-b013] Hendrickson H, Lawrence JG (2006). Selection for chromosome architecture in bacteria. J Mol Evol.

[pgen-0030226-b014] Coskun-Ari FF, Hill TM (1997). Sequence-specific interactions in the Tus-Ter complex and the effect of base pair substitutions on arrest of DNA replication in Escherichia coli. J Biol Chem.

[pgen-0030226-b015] Horiuchi T, Fujimura Y (1995). Recombinational rescue of the stalled DNA replication fork: a model based on analysis of an Escherichia coli strain with a chromosome region difficult to replicate. J Bacteriol.

[pgen-0030226-b016] Sharma B, Hill TM (1995). Insertion of inverted Ter sites into the terminus region of the Escherichia coli chromosome delays completion of DNA replication and disrupts the cell cycle. Mol Microbiol.

[pgen-0030226-b017] Bidnenko V, Ehrlich SD, Michel B (2002). Replication fork collapse at replication terminator sequences. EMBO J.

[pgen-0030226-b018] Bidnenko V, Lestini R, Michel B (2006). The Escherichia coli UvrD helicase is essential for Tus removal during recombination-dependent replication restart from Ter sites. Mol Microbiol.

[pgen-0030226-b019] Nielsen HJ, Li Y, Youngren B, Hansen FG, Austin S (2006). Progressive segregation of the Escherichia coli chromosome. Mol Microbiol.

[pgen-0030226-b020] Nielsen HJ, Ottesen JR, Youngren B, Austin SJ, Hansen FG (2006). The Escherichia coli chromosome is organized with the left and right chromosome arms in separate cell halves. Mol Microbiol.

[pgen-0030226-b021] Teleman AA, Graumann PL, Lin DC, Grossman AD, Losick R (1998). Chromosome arrangement within a bacterium. Curr Biol.

[pgen-0030226-b022] Viollier PH, Thanbichler M, McGrath PT, West L, Meewan M (2004). Rapid and sequential movement of individual chromosomal loci to specific subcellular locations during bacterial DNA replication. Proc Natl Acad Sci U S A.

[pgen-0030226-b023] Wang X, Liu X, Possoz C, Sherratt DJ (2006). The two Escherichia coli chromosome arms locate to separate cell halves. Genes Dev.

[pgen-0030226-b024] Niki H, Yamaichi Y, Hiraga S (2000). Dynamic organization of chromosomal DNA in Escherichia coli. Genes Dev.

[pgen-0030226-b025] Valens M, Penaud S, Rossignol M, Cornet F, Boccard F (2004). Macrodomain organization of the Escherichia coli chromosome. EMBO J.

[pgen-0030226-b026] Yamaichi Y, Niki H (2004). migS, a cis-acting site that affects bipolar positioning of oriC on the Escherichia coli chromosome. EMBO J.

[pgen-0030226-b027] Perals K, Cornet F, Merlet Y, Delon I, Louarn JM (2000). Functional polarization of the Escherichia coli chromosome terminus: the dif site acts in chromosome dimer resolution only when located between long stretches of opposite polarity. Mol Microbiol.

[pgen-0030226-b028] Bigot S, Sivanathan V, Possoz C, Barre FX, Cornet F (2007). FtsK, a literate chromosome segregation machine. Mol Microbiol.

[pgen-0030226-b029] Lesterlin C, Barre FX, Cornet F (2004). Genetic recombination and the cell cycle: what we have learned from chromosome dimers. Mol Microbiol.

[pgen-0030226-b030] Rebollo JE, Francois V, Louarn JM (1988). Detection and possible role of two large nondivisible zones on the Escherichia coli chromosome. Proc Natl Acad Sci U S A.

[pgen-0030226-b031] Konrad EB (1977). Method for the isolation of Escherichia coli mutants with enhanced recombination between chromosomal duplications. J Bacteriol.

[pgen-0030226-b032] Segall A, Mahan MJ, Roth JR (1988). Rearrangement of the bacterial chromosome: forbidden inversions. Science.

[pgen-0030226-b033] Hill CW, Gray JA (1988). Effects of chromosomal inversion on cell fitness in Escherichia coli K-12. Genetics.

[pgen-0030226-b034] Miesel L, Segall A, Roth JR (1994). Construction of chromosomal rearrangements in Salmonella by transduction: inversions of non-permissive segments are not lethal. Genetics.

[pgen-0030226-b035] Guijo MI, Patte J, del Mar Campos M, Louarn JM, Rebollo JE (2001). Localized remodeling of the Escherichia coli chromosome: the patchwork of segments refractory and tolerant to inversion near the replication terminus. Genetics.

[pgen-0030226-b036] Campo N, Dias MJ, Daveran-Mingot ML, Ritzenthaler P, Le Bourgeois P (2004). Chromosomal constraints in Gram-positive bacteria revealed by artificial inversions. Mol Microbiol.

[pgen-0030226-b037] Lesterlin C, Mercier R, Boccard F, Barre FX, Cornet F (2005). Roles for replichores and macrodomains in segregation of the Escherichia coli chromosome. EMBO Rep.

[pgen-0030226-b038] Louarn JM, Kuempel PL, Cornet F, Higgins NP (2005). The terminus region of the E. coli chromosome, or, all's well that ends well. The bacterial chromosome.

[pgen-0030226-b039] Huisman O, D'Ari R, Gottesman S (1984). Cell-division control in Escherichia coli: specific induction of the SOS function SfiA protein is sufficient to block septation. Proc Natl Acad Sci U S A.

[pgen-0030226-b040] Mirkin EV, Castro Roa D, Nudler E, Mirkin SM (2006). Transcription regulatory elements are punctuation marks for DNA replication. Proc Natl Acad Sci U S A.

[pgen-0030226-b041] French S (1992). Consequences of replication fork movement through transcription units in vivo. Science.

[pgen-0030226-b042] Mirkin EV, Mirkin SM (2005). Mechanisms of transcription-replication collisions in bacteria. Mol Cell Biol.

[pgen-0030226-b043] Cornet F, Louarn J, Patte J, Louarn JM (1996). Restriction of the activity of the recombination site dif to a small zone of the Escherichia coli chromosome. Genes Dev.

[pgen-0030226-b044] Corre J, Louarn JM (2005). Extent of the activity domain and possible roles of FtsK in the Escherichia coli chromosome terminus. Mol Microbiol.

[pgen-0030226-b045] Espeli O, Levine C, Hassing H, Marians KJ (2003). Temporal regulation of topoisomerase IV activity in E. coli. Mol Cell.

[pgen-0030226-b046] Lin DC, Grossman AD (1998). Identification and characterization of a bacterial chromosome partitioning site. Cell.

[pgen-0030226-b047] de Boer PA, Crossley RE, Rothfield LI (1989). A division inhibitor and a topological specificity factor coded for by the minicell locus determine proper placement of the division septum in E. coli. Cell.

[pgen-0030226-b048] Bernhardt TG, de Boer PA (2005). SlmA, a nucleoid-associated, FtsZ binding protein required for blocking septal ring assembly over chromosomes in E. coli. Mol Cell.

[pgen-0030226-b049] Moulin L, Rahmouni AR, Boccard F (2005). Topological insulators inhibit diffusion of transcription-induced positive supercoils in the chromosome of Escherichia coli. Mol Microbiol.

[pgen-0030226-b050] Olsson JA, Nordstrom K, Hjort K, Dasgupta S (2003). Eclipse-synchrony relationship in Escherichia coli strains with mutations affecting sequestration, initiation of replication and superhelicity of the bacterial chromosome. J Mol Biol.

