# Supplementary material for: Chromosome Structuring Limits Genome Plasticity in Escherichia coli
Source: PLoS Genet. 2007 Dec 14;3(12):e226. doi: 10.1371/journal.pgen.0030226 (PMC2134941; doi:10.1371/journal.pgen.0030226)
Supplement: Supplementary file 1 [file pgen.0030226.sd001.pdf]

Supplementary text for Chromosome structuring limits genome plasticity in *E. coli*

Ter sites impede replication forks with various efficiencies

To estimate the respective strength of Ter sites *in vivo* in a strain producing wt levels of Tus, we have generated strains where different Ter sites are inverted. Strains carry a region bordered by two Ter sites that block both replication forks (for example between TerE and TerD in Figure S1). We selected TerE as a predicted very strong site (strains Intra R1 and Intra R2 in Table1), the couple TerH and TerI (TerHI, strains Intra R-NS^{right}1 to 4 in Table 1) as a predicted moderately strong site, and TerI (strain Intra R-NS^{right}3 ΔTerH in Table 1) or TerJ (strain L-NS^{left}1 in Table 1) as a predicted weak site. As observed before with strong ectopic TerA site [1-3], the inversion of the strong TerE site severely affected colony formation (Figure 2B). The size of colonies containing inverted TerHI sites was significantly reduced whereas the inversion of only a weak site, TerI or TerJ, had little effect. The detrimental effect produced by the inversions of TerE or TerHI can be suppressed either by a deletion of the *tus* gene or by the precise deletion of the Ter sites present in the inverted fragment (Figure S1B).

The presence of an inverted TerE site is highly detrimental and overnight cultures accumulated variants that likely acquired genetic suppressors restoring normal growth (data not shown). It was therefore difficult to measure the defect of such inversions over 60 generations. In contrast, the relative stability of strains carrying TerHI in an inverted configuration allowed measures of the growth over 60 generations (Figure S1C): a significant defect was associated with the moderately strong site as the ratio of inverted to wt cells was below 0.01 after 60 generations.

As previously observed [1-3], we found that RecA was essential to obtain viable colonies after inversion of a strong site, TerE (Figure S1B). By contrast, colonies could be

obtained in a *recA* background after inversion of the moderately strong TerHI sites even though they show severe growth defects compared to the wt configuration (Figure S1B). Finally, inversion of TerI or TerJ has little effect in a *recA* background (Figure S1B).

The effects of inverting TerE carrying region was tested in different genetic backgrounds. Recombinants could be obtained in a *recFOR* background but, as predicted from previous results with ectopic TerA site [1-4], not in conditions inhibiting either RecBC DNA recombination or SOS induction, i.e. in *recBC*, *lexA ind-* (SOS- Rec+) or *recA* $lexA^{def}$ (Rec- SOS+) genetic backgrounds (Table 1). It should be noted that the absence of recombinant colonies resulted from lethality as recombination at the DNA level was detected (data not shown). The absence of viable recombinants in these four genetic backgrounds indicates that two RecA actions are required for promoting RecBC-dependent recombination and induction of the SOS response (Figure S1D). As expected, the requirement for RecA is suppressed by the deletion of TerE or by the deletion of *tus* (data not shown).

The microscopic observation of cells with inverted TerE revealed the occurrence of filamentation with DNA accumulating in non-segregated nucleoids (Figure S1E and S1F). 43% of abnormal cells were detected in the inverted configuration: 24% consisted of more or less elongated cells with un-segregated nucleoids and 19% of cells with a *par* phenotype, i.e. cells containing a compacted nucleoid and a large zone free of DNA similar to those found in partition defective mutants [5]. The inability to obtain viable recombinant colonies in a *lexA ind-* background indicated the essentiality of the SOS response in the presence of Ter sites impeding progression of both replication forks. The presence in these cells of a plasmid carrying a *gfp* gene under the control of the P_{SfiA} promoter allowed the direct visualization of the SOS induction (Figure S1F and Figure S2). This induction was quantified using a P_{sfiA}-*uidA* fusion; β-glucoronidase activity was 6-fold higher than in the non-inverted control (Table 1), a high level similar to that obtained with non-inverted cells after UV irradiation

with a dose of 40 J/m^2 (data not shown). In the same conditions, the inversion of the moderately strong TerHI site induced the SOS response only two to three fold (Table 1).

Altogether these results indicate that efficiency of replication arrest at different chromosomal Ter sites correlates with the predictions based on *in vitro* affinities and on replication arrest activity of TerB mutant sites. They show that in conditions of wt level of Tus protein, blocking the two forks by the strong TerE site renders RecBCD-dependent recombination as previously observed with TerA [2]. SOS induction is also essential in these conditions. The effect of the moderate site TerH in the inverted orientation was less severe but still significant. Inverted weak TerI and TerJ sites do not appear to affect viability suggesting they do not significantly impede replication.

Intra-replichore inversion that intermingle Ori and Right MDs are detrimental

When tested in different genetic backgrounds, the proximal combinations could not give rise to viable recombinants in a *recA*, *recBC*, *lexA ind-* background indicating that SOS induction and possibly homologous recombination were required for viability. The measures of SOS response using a P_{sfiA}-*uidA* gene fusion indicated that the level of induction was less than two-fold higher than in the wt configuration (Table 2).

Since the inverted terHI sites could be responsible for SOS induction as observed in inversions between the NS^{right} region and the Right MD (control c in Figure 5A), we deleted these two sites successively. In the presence of the unique TerI site (ΔTerH in Figure 5C), viable recombinant colonies could be obtained when the inverted segment extended from the Right MD to 95' in the Ori MD and the inversion only slightly affected colony formation (strains intra O-R1 to 3 in Figure 5C). However, in a *recA* background, the inversion of segments between Ori and Right MDs strongly delayed colony formation (strains intra O-R1 to 3 in Figure 5C) whereas inversions of segments between NS^{right} and Right MD had a minor

effect (strain c in Figure 5C). In the absence of both TerH and TerI (ΔTerHI in Figure 5C), viable recombinant colonies could be obtained when the inverted segment extended from the Right MD to 95' (strains intra O-R1 to 3 in Figure 5C). In a *recA* background, recombinant colonies were less affected than when TerI was present (Figure 5C). Therefore, the effect of TerI in the absence of *recA,* which was very modest in inversion between the NS^{right} region and the Right MD seemed to be important when associated to an inversion involving Right and Ori MDs. Altogether the results indicate that, for reasons that remain to be understood, the presence of inverted TerH and TerI even in a *recA*+ context has a dramatic effect when associated to inversions combining Ori and Right MDs.

Distal inversions remained lethal when TerH or both TerH and TerI were deleted (Figure 5D and data not shown, strains Intra O-R6 to 8 in Table 2). Remarkably, viable colonies could be obtained on minimal growth medium (Figure 5D). These recombinants showed growth defects in minimal medium supplemented with casamino-acids (Figure 5D) and could not be propagated in rich medium (data not shown).

Construction of strains

Most of the strains that support recombination between *att*B and *att*P or between *att*L and *att*R have been isolated in a previous study [6]. New combinations of *att*L and *att*R sites have been obtained by bacteriophage P1 transduction using strains from different collections. Strains FBG150-*att*L_{23} and FBG150-*att*L_{35}, were obtained by conjugative transposition of pUT-Tn*5*-*att*L in FBG150 as described before [6]. Strain FBG151 and FBG152 have been obtained by using pI-pKO3-attR, a pKO3 derivative [7] carrying the *aadA* gene, *att*R fused to the 3' part of *lacZ* between "*ybhC*" and "*ybhB*". In strain FBG151, orientation of *att*R (clockwise) is opposite to that of strain FBG152 (counterclockwise). Strains FBG161 and FBG162 have been obtained by using pI-pKO3-*att*L, a pKO3 derivative [7] carrying the *aadA*

gene, *att*L fused to the 5' part of *lacZ* between "*ybhC*" and "*ybhB*". In strain FBG162, orientation of *att*L (clockwise) is opposite to that of strain FBG161 (counterclockwise).

Replacement of *tus* by *frt-cat-frt* was performed by *in vivo* recombination in DY330 [8] of a PCR fragment generated by PCR amplification using as template plasmid pKD3 and oligos 5′-GCTATAAAATAAGTATGTTGTAACTAAAGTGGTTAATATTGTGTAGGCTGGAGCTGCTTC-3′ and 5′-TTAATCTGCAACATACAGGTGCAGCCGTGGAATGATCAAACATATGAATATCCTCCTTAG-3′. Replacement of *tus* by a *tetR* gene was made by substituting the *Bcl*I fragment of *tus* by a *Bam*HI fragment carrying *tetR* obtained from pNK2883 [9]. The Δ*tus*::*tetR* fragment was cloned in pKO3 and inserted at the *tus* locus as described [7]. Deletion of Ter sites was performed by *in vivo* recombination in DY330 of PCR fragments generated by PCR amplification using as template plasmid pKD3 and oligos 5′- TTCGCGGTCTTGATGGCCTGTAATTTTTAAAAAAAATCCGATTGTGTAGGCTGGAG-3′ and 5′-TTTCTGCGCCGTCGTTGCCGGAGCACAATGGAACATTAATATATGAATATCCTCCTTAG-3′ (TerA), 5′-TATCCTTTGCGCGGCATGATGTCGCGCTTTTTTTATGCGTATTGTGTAGGCTGGAG-3′ and 5′-TAATGGCTCTTAATGAAATCCGGCGAAATAAACCATATAAATATGAATATCCTCCTTAG-3′ (TerD), 5′-GCGACAGCGTCTCGCTCCGCGTAACAAATACGACGCAAACTCTGTGTAGGCTGGAG-3′ and 5′-GCGTTAGGCGGAAGTTAATGGTCATGACGAGACTATCCCCATATGAATATCCTCCTTAG-3′ (TerE), 5′-GTGATCGCCGCCGTGAAGGACAAACTGGAAACGCTGAAAGTGTAGGCTGGAG-3′ and 5′-CGCCCACCACAATAAACTCTTCCAGCAACTTGCCGCTGAGATATGAATATCCTCCTTAG-3′ (TerH), 5′-GATAGTGGCGTATTGCTGGAACGGTTGATTGCGCAAAACCCGATTGTGTAGGCTGGAG-3′ and 5′-CCGGGCTGGCCCCCAGCAGGACGCCACCATCAGCCAGCGGATATGAATATCCTCCTTAG-3′ (TerI). Deletion of the *sfiA* gene was performed by *in vivo* recombination in DY330 of a PCR fragment generated by PCR amplification using as template plasmid pKD3 and oligos 5′-ATGTACACTTAGGCTATGCACATCGTTCTTCGTCGTTCTCAGTGTAGGCTGGAGCTGCTTC-3′ and 5′-TTAATGATACAAATTAGAGTGAATTTTTAGCCCGGAAAGTTGCATATGAATATCCTCCTTAG-3′. *dif* insertion in the new replichore junction of strain Intra R-T2 Δ*dif* was performed by cloning a 275 bp *Nhe*I PCR

fragment carrying *dif* (coordinates 1588632-1588901) in pI-pKO3-*att*R and insertion of this region between "*ybhC*" and "*ybhB*".

Different mutant alleles were transferred by bacteriophage P1 transduction. When necessary, the *cat* gene flanked by *frt* sites was deleted by expressing FLP from pCP20 [10].

Construction of plasmids

Plasmid pI-pKO3-*att*L and pI-pKO3-*att*L^{off} have been obtained by cloning a *Sal*I fragment of pG-attL-attR [6] carrying *att*L fused to the 5' part of *lacZ*.

Plasmid pBAD-P_{sfiA}-*uidA* was constructed by cloning the *sfiA* promoter in plasmid pBAD-LG4 [11] from which *lacZ* was deleted by a *Hin*dIII-*Xho*I deletion followed by a treatment with Klenow fragment. A x bp Acc65I-NsiI PCR fragment carrying the sfiA promoter was inserted in pBAD-LG4-ΔlacZ digested by . Plasmid pTSC29-CXI was obtained by cloning a x-y fragment carrying cI857-PR-xis-int from pTSA29-CXI in pTSC29 [12]. pZA-P_{sfiA}-*gfp* was obtained by cloning a *Bgl*II-*Bam*HI PCR fragment carrying P_{sfiA} fused to *gfp* in pZA31-MCS Δ*Xho*I. The PCR fragment was generated using as template plasmid pFX378 (a colE1 derivative carrying P_{sfiA} fused to *gfp*; F.-X. Barre, personnal gift) and oligos `5'-GGGAAAAGATCTGTTAACTACGAAAATAGGCA-3'` and `5'-GGGAAAGGATCCACTAGTTTATTTGTATGGTT-3'`.

References

1. Bidnenko V, Ehrlich SD, Michel B (2002) Replication fork collapse at replication terminator sequences. Embo J 21: 3898-3907.

2. Horiuchi T, Fujimura Y (1995) Recombinational rescue of the stalled DNA replication fork: a model based on analysis of an *Escherichia coli* strain with a chromosome region difficult to replicate. J Bacteriol 177: 783-791.

3. Sharma B, Hill TM (1995) Insertion of inverted Ter sites into the terminus region of the *Escherichia coli* chromosome delays completion of DNA replication and disrupts the cell cycle. Mol Microbiol 18: 45-61.

4. Bidnenko V, Lestini R, Michel B (2006) The *Escherichia coli* UvrD helicase is essential for Tus removal during recombination-dependent replication restart from Ter sites. Mol Microbiol 62: 382-396.

5. Kato J, Nishimura Y, Yamada M, Suzuki H, Hirota Y (1988) Gene organization in the region containing a new gene involved in chromosome partition in Escherichia coli. J Bacteriol 170: 3967-3977.

6. Valens M, Penaud S, Rossignol M, Cornet F, Boccard F (2004) Macrodomain organization of the *Escherichia coli* chromosome. Embo J 23: 4330-4341.

7. Link AJ, Phillips D, Church GM (1997) Methods for generating precise deletions and insertions in the genome of wild-type Escherichia coli: application to open reading frame characterization. J Bacteriol 179: 6228-6237.

8. Yu D, Ellis HM, Lee EC, Jenkins NA, Copeland NG, et al. (2000) An efficient recombination system for chromosome engineering in Escherichia coli. Proc Natl Acad Sci U S A 97: 5978-5983.

9. Kleckner N, Bender J, Gottesman S (1991) Uses of transposons with emphasis on Tn10. Methods Enzymol 204: 139-180.

10. Cherepanov PP, Wackernagel W (1995) Gene disruption in Escherichia coli: TcR and KmR cassettes with the option of Flp-catalyzed excision of the antibiotic-resistance determinant. Gene 158: 9-14.

11. Moulin L, Rahmouni AR, Boccard F (2005) Topological insulators inhibit diffusion of transcription-induced positive supercoils in the chromosome of *Escherichia coli*. Mol Microbiol 55: 601-610.

12. Phillips GJ (1999) New cloning vectors with temperature-sensitive replication. Plasmid 41: 78-81.
