# Supplementary material for: Chromosome Structuring Limits Genome Plasticity in Escherichia coli
Source: PLoS Genet. 2007 Dec 14;3(12):e226. doi: 10.1371/journal.pgen.0030226 (PMC2134941; doi:10.1371/journal.pgen.0030226)
Supplement: Supplementary file 2 [file pgen.0030226.sg001.pdf]

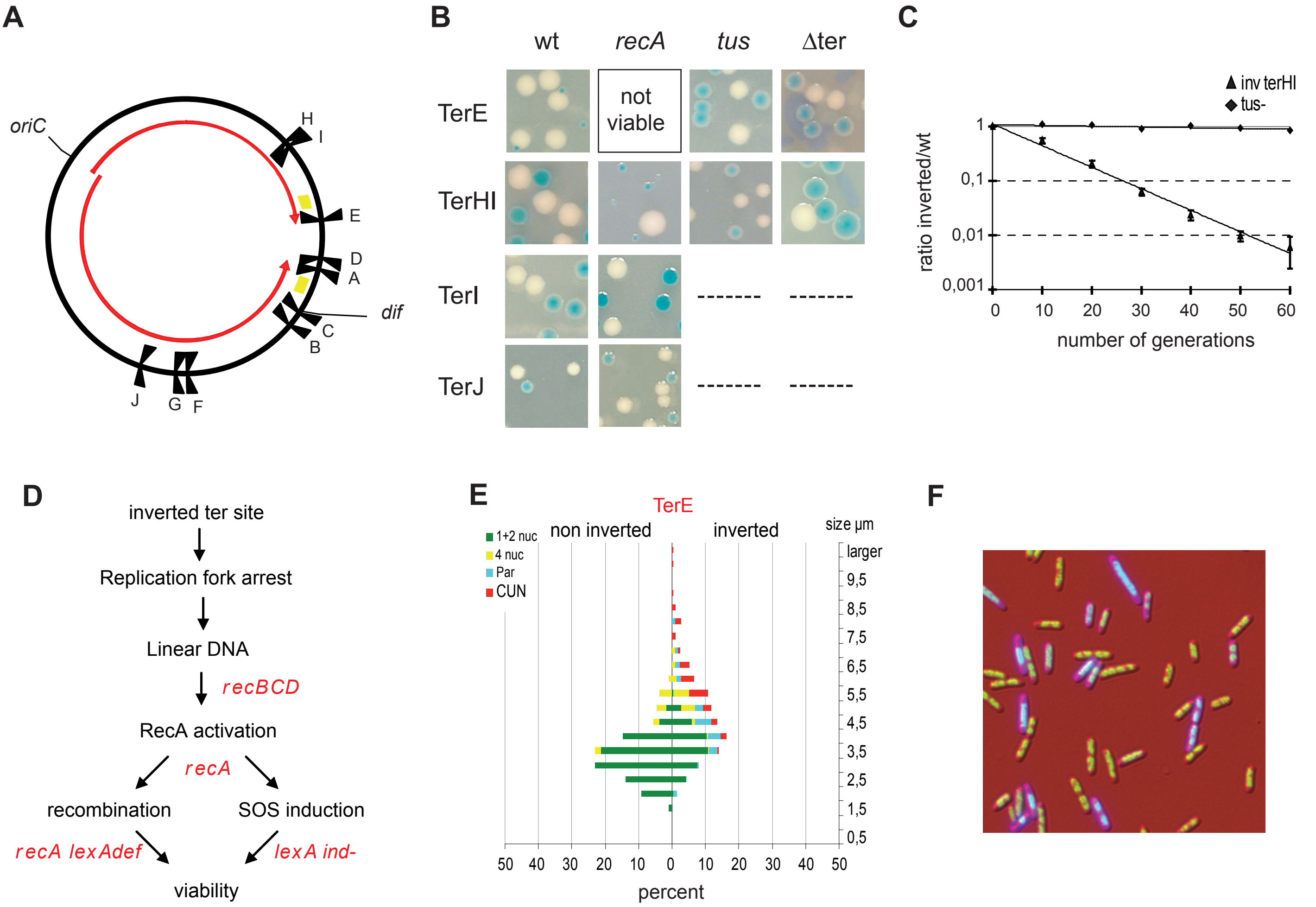
A
oriC
H
I
E
D
A
dif
C
B
J
G
F
B
wt
recA
tus
Δter
TerE
not viable
TerHI
TerI
TerJ
C
inv terHI
tus-
ratio inverted/wt
1
0,1
0,01
0,001
0
10
20
30
40
50
60
number of generations
D
inverted ter site
Replication fork arrest
Linear DNA
recBCD
RecA activation
recA
recombination
SOS induction
recA lexAdef
lexA ind-
viability
E
TerE
1+2 nuc
4 nuc
Par
CUN
non inverted
inverted
size µm
larger
9,5
8,5
7,5
6,5
5,5
4,5
3,5
2,5
1,5
0,5
50 40 30 20 10 0 10 20 30 40 50
percent
F

Figure S1
