# Supplementary material for: Chromosome Structuring Limits Genome Plasticity in Escherichia coli
Source: PLoS Genet. 2007 Dec 14;3(12):e226. doi: 10.1371/journal.pgen.0030226 (PMC2134941; doi:10.1371/journal.pgen.0030226)
Supplement: Supplementary file 3 [file pgen.0030226.sg002.pdf]

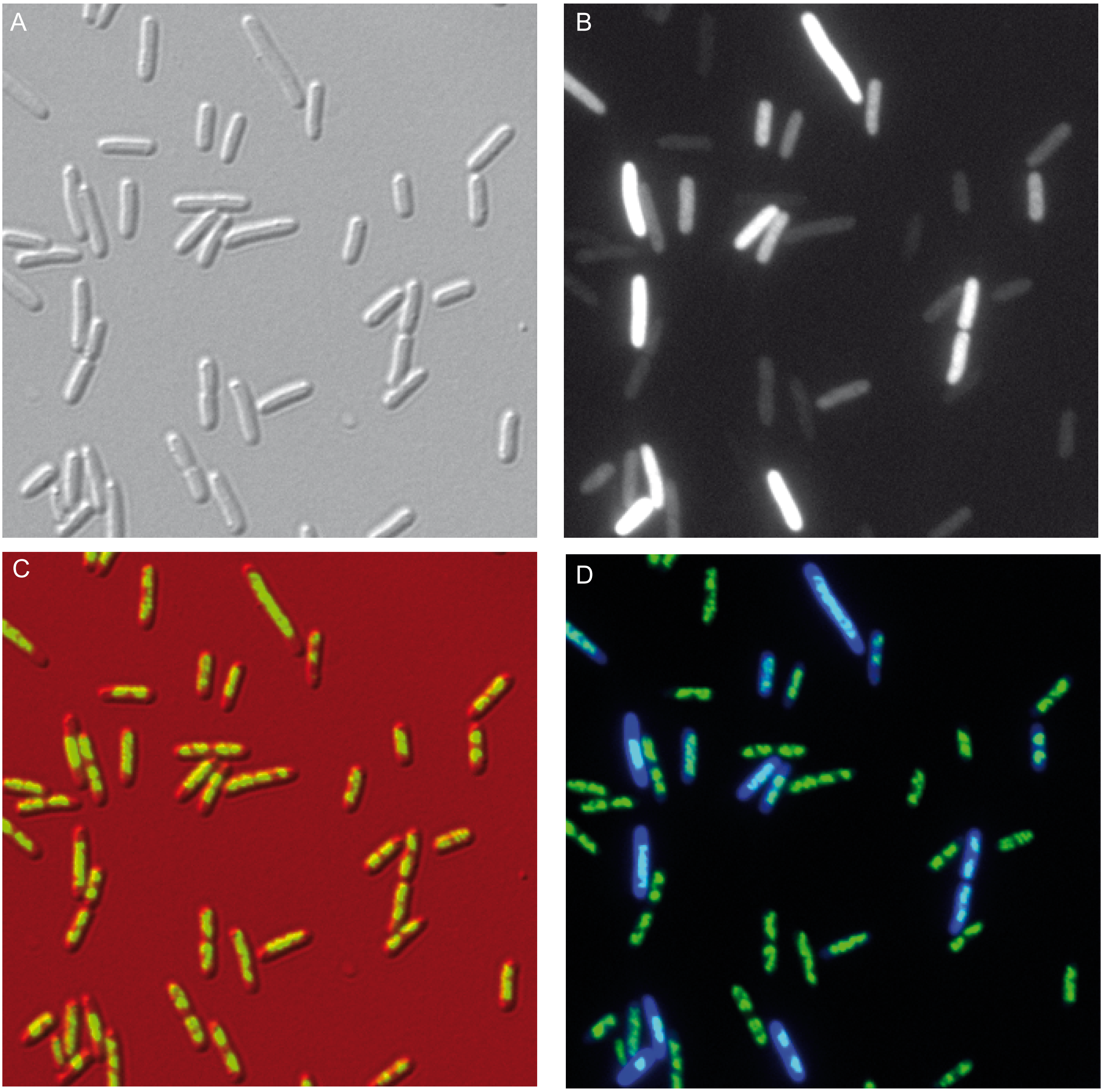

Figure S2
