# Supplementary material for: Chromosome Structuring Limits Genome Plasticity in Escherichia coli
Source: PLoS Genet. 2007 Dec 14;3(12):e226. doi: 10.1371/journal.pgen.0030226 (PMC2134941; doi:10.1371/journal.pgen.0030226)
Supplement: Supplementary file 4 [file pgen.0030226.sg003.pdf]

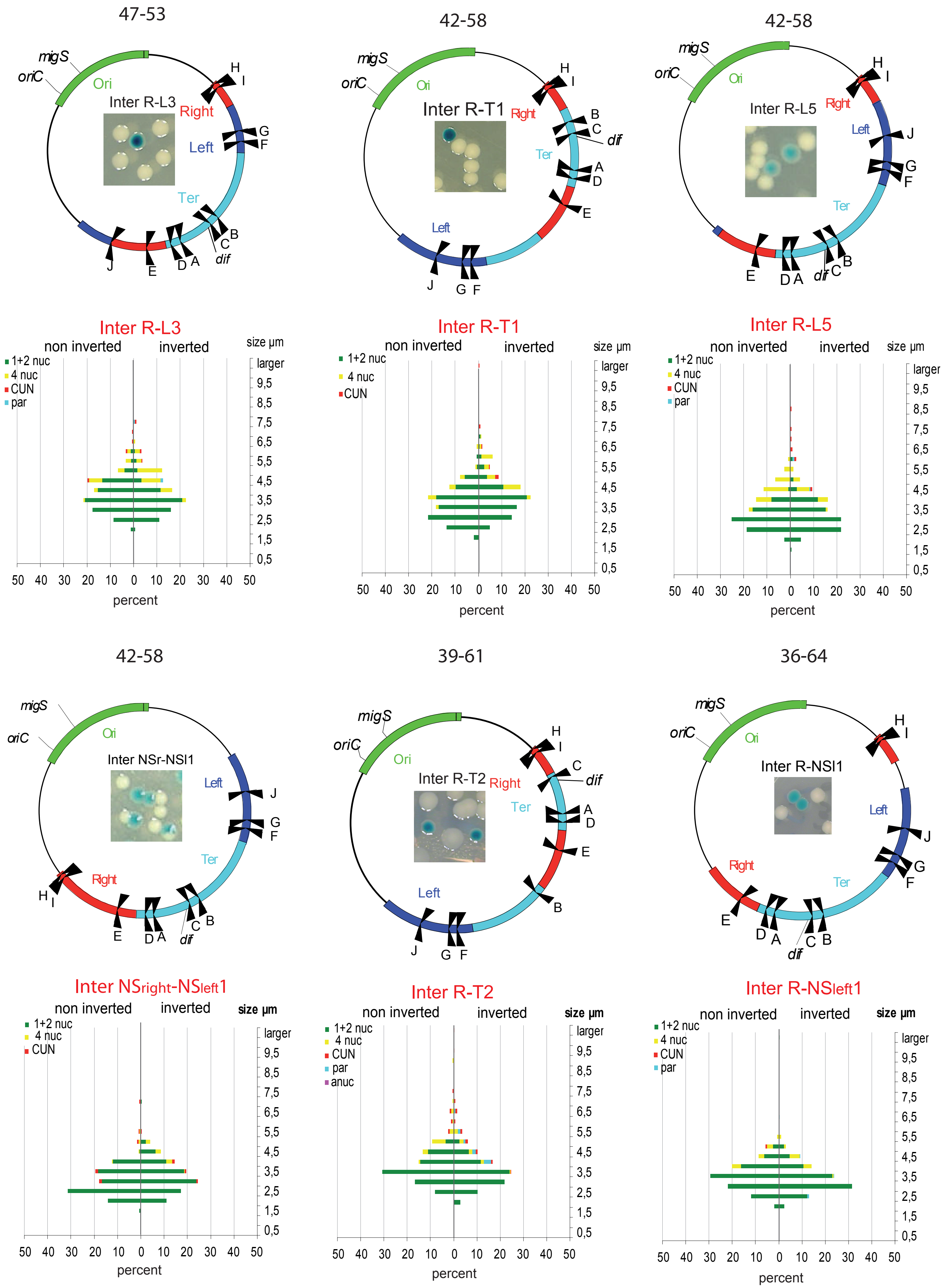

47-53
42-58
42-58
migS
oriC
Ori
Inter R-L3
Right
Left
Ter
H
I
G
F
B
C
dif
J
E
D
A
Inter R-T1
Inter R-L5
Inter R-L3
non inverted
inverted
size µm
1+2 nuc
4 nuc
CUN
par
larger
9,5
8,5
7,5
6,5
5,5
4,5
3,5
2,5
1,5
0,5
50 40 30 20 10 0 10 20 30 40 50
percent
Inter R-T1
Inter R-L5
42-58
39-61
36-64
Inter NSr-NSl1
Inter R-T2
Inter R-NSl1
Inter NSright-NSleft1
Inter R-T2
anuc
Inter R-NSleft1

Figure S3A

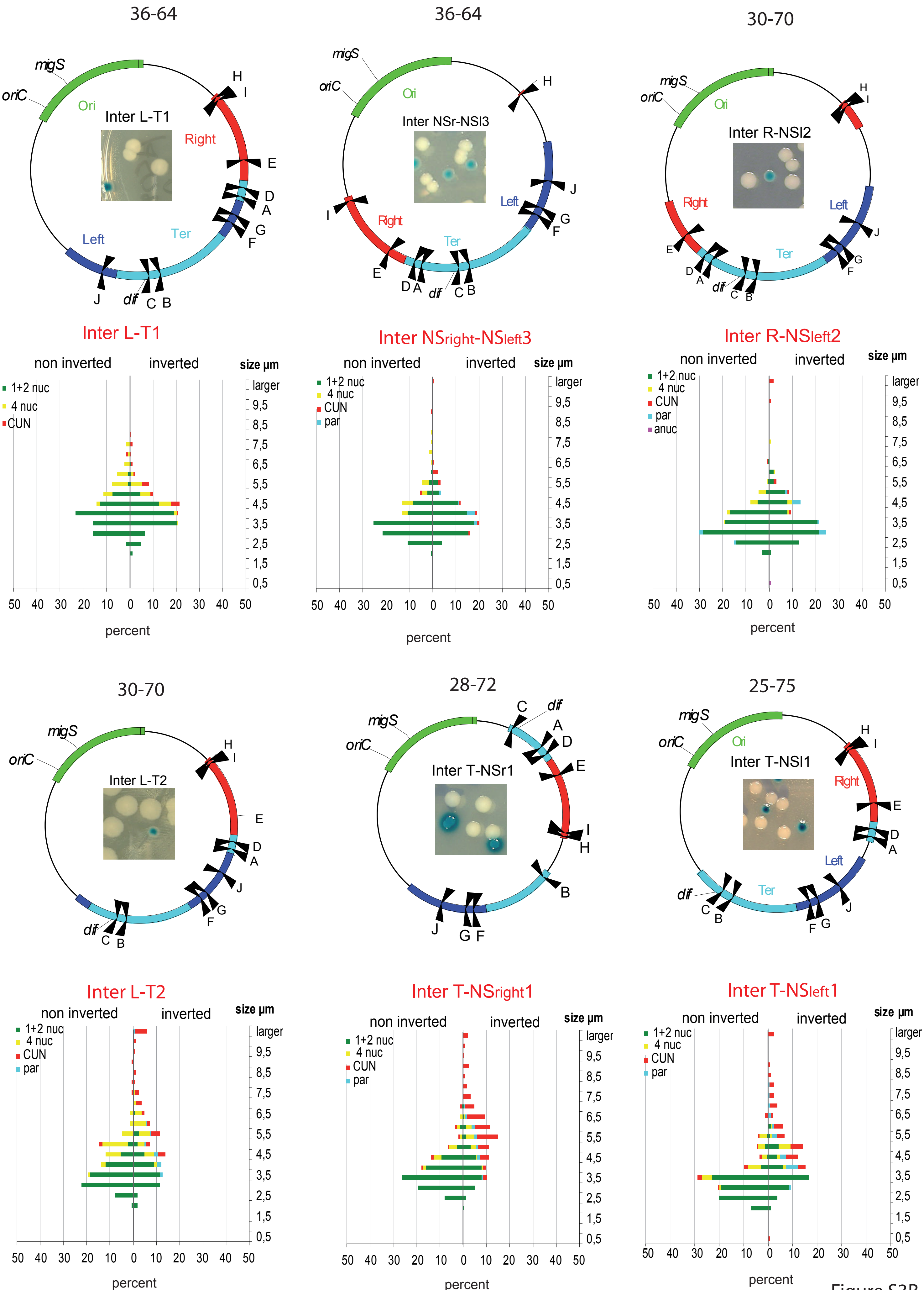
36-64
36-64
30-70
migS
oriC
Ori
Inter L-T1
Right
Left
Ter
H
I
E
D
A
G
F
J
dif
C
B
Inter NSr-NSl3
Inter R-NSl2
Inter L-T1
Inter NSright-NSleft3
Inter R-NSleft2
non inverted
inverted
size µm
1+2 nuc
4 nuc
CUN
par
anuc
larger
9,5
8,5
7,5
6,5
5,5
4,5
3,5
2,5
1,5
0,5
50 40 30 20 10 0 10 20 30 40 50
percent
30-70
28-72
25-75
Inter L-T2
Inter T-NSr1
Inter T-NSl1
Inter L-T2
Inter T-NSright1
Inter T-NSleft1

Figure S3B

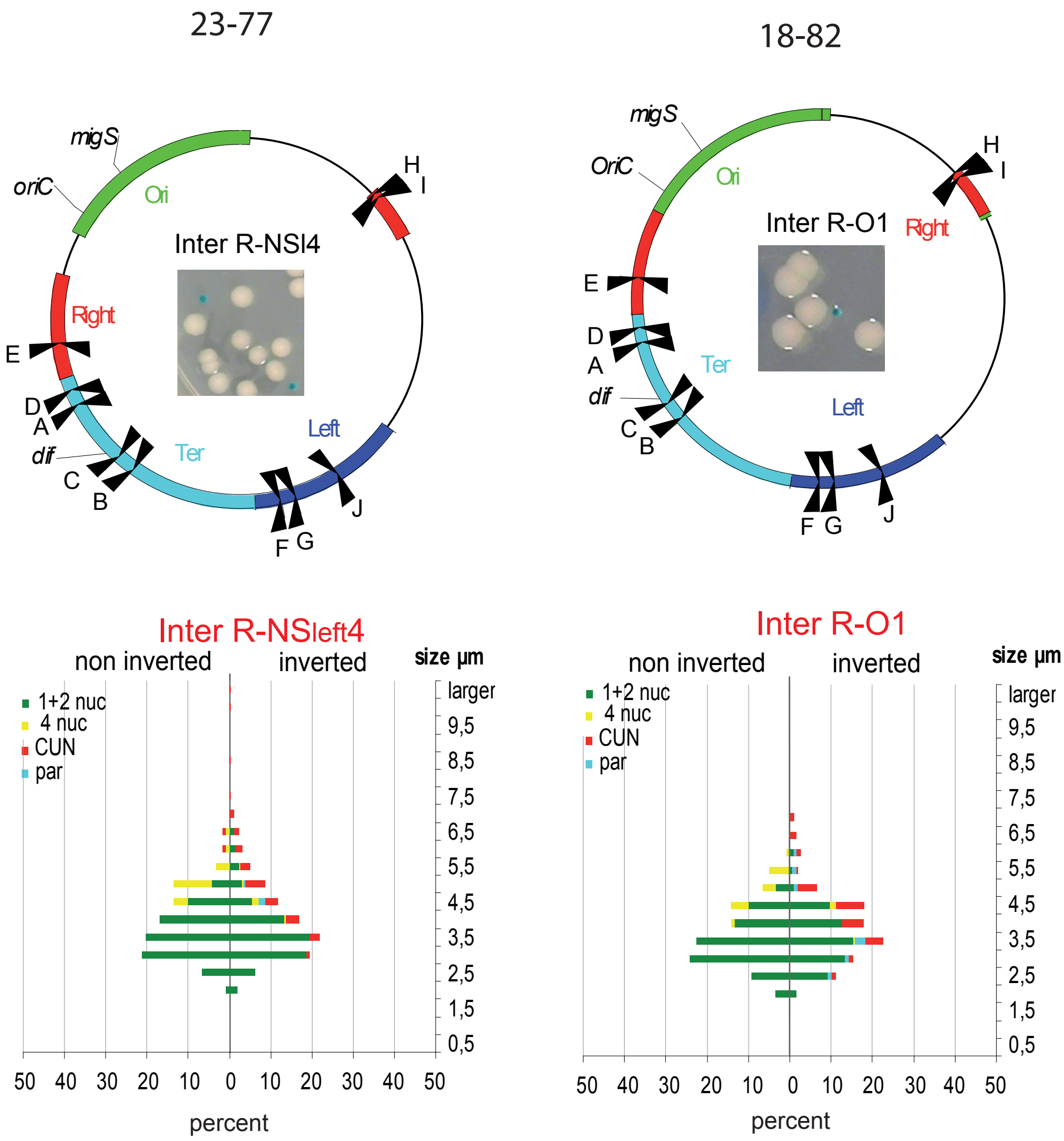
23-77
18-82
migS
oriC
Ori
Inter R-NSl4
Right
Ter
Left
dif
A
B
C
D
E
F
G
H
I
J
OriC
Inter R-O1
Inter R-NSleft4
Inter R-O1
non inverted
inverted
size µm
1+2 nuc
4 nuc
CUN
par
larger
9,5
8,5
7,5
6,5
5,5
4,5
3,5
2,5
1,5
0,5
50 40 30 20 10 0 10 20 30 40 50
percent

Figure S3C
