# Supplementary material for: Chromosome Structuring Limits Genome Plasticity in Escherichia coli
Source: PLoS Genet. 2007 Dec 14;3(12):e226. doi: 10.1371/journal.pgen.0030226 (PMC2134941; doi:10.1371/journal.pgen.0030226)
Supplement: Supplementary file 5 [file pgen.0030226.sg004.pdf]

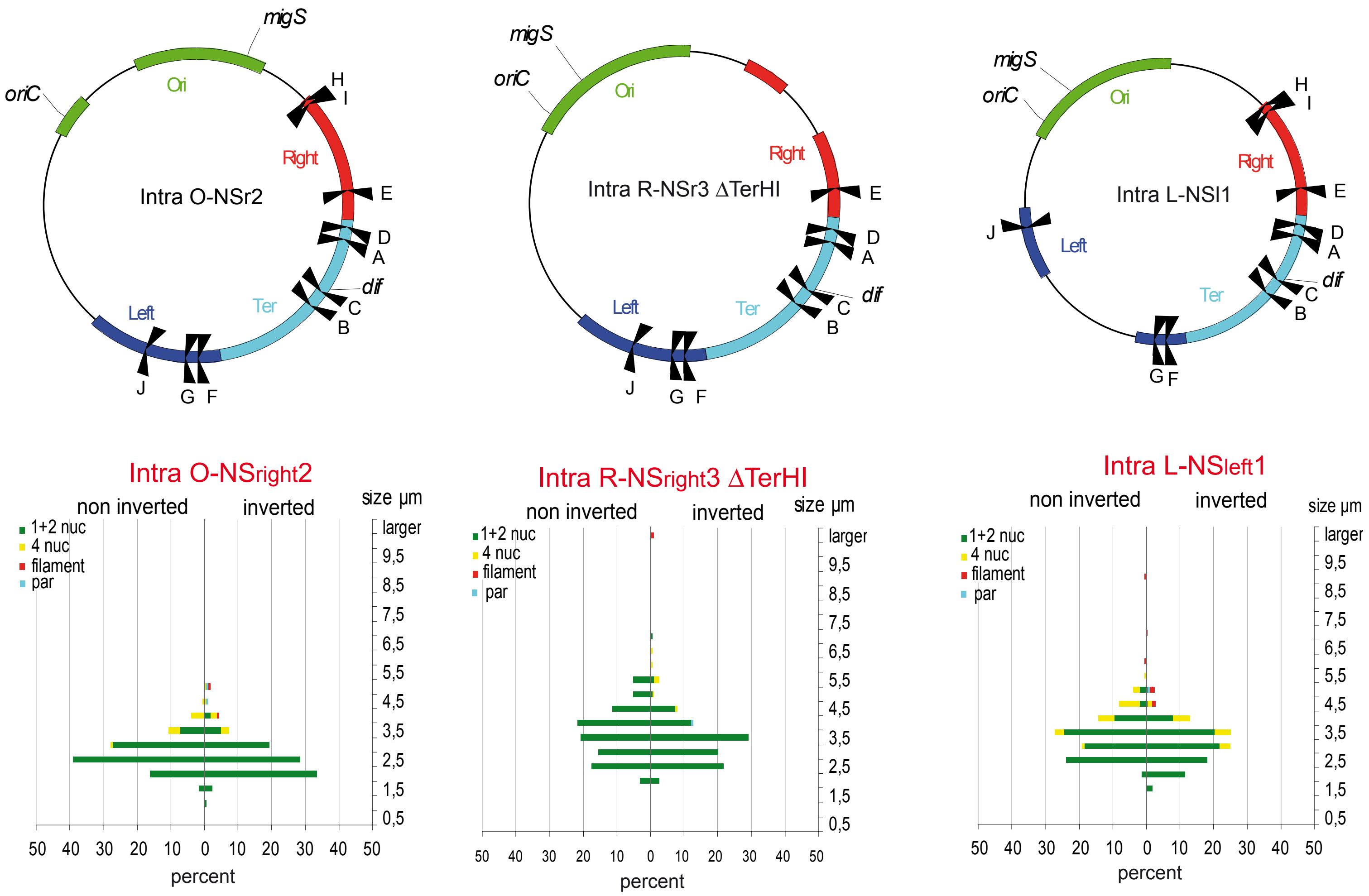

migS
oriC
Ori
Right
Left
Ter
dif
H
I
E
D
A
C
B
J
G
F
Intra O-NSr2
Intra R-NSr3 ΔTerHI
Intra L-NSl1
Intra O-NSright2
Intra R-NSright3 ΔTerHI
Intra L-NSleft1
non inverted
inverted
size µm
1+2 nuc
4 nuc
filament
par
larger
9,5
8,5
7,5
6,5
5,5
4,5
3,5
2,5
1,5
0,5
50 40 30 20 10 0 10 20 30 40 50
percent

Figure S4
