# Supplementary material for: Chromosome Structuring Limits Genome Plasticity in Escherichia coli
Source: PLoS Genet. 2007 Dec 14;3(12):e226. doi: 10.1371/journal.pgen.0030226 (PMC2134941; doi:10.1371/journal.pgen.0030226)
Supplement: Supplementary file 7 [file pgen.0030226.sg006.pdf]

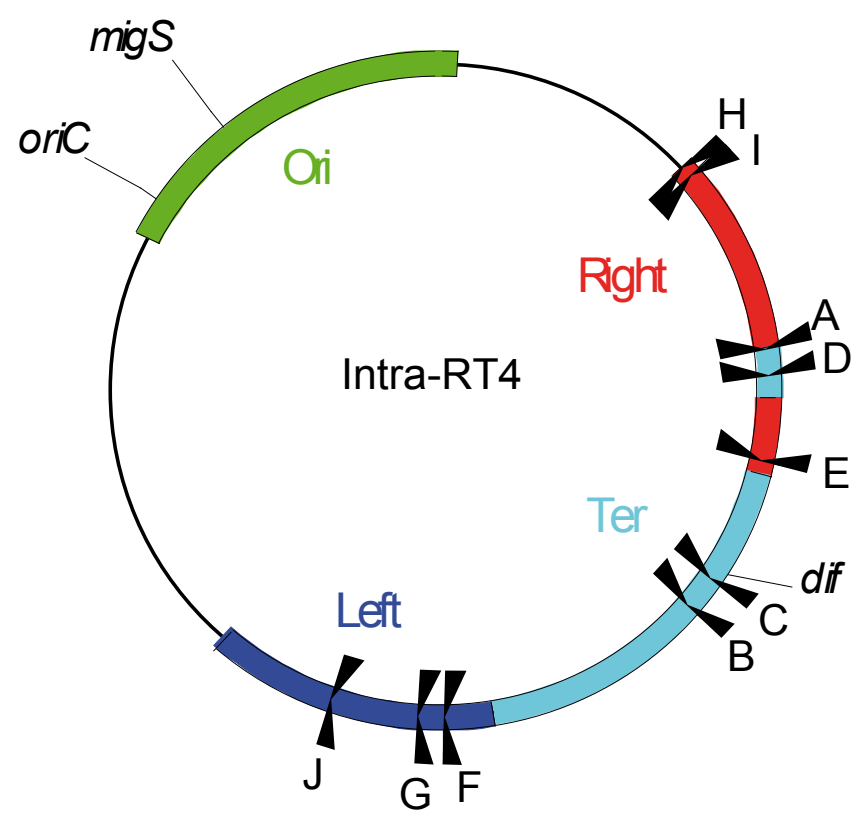
migS
oriC
Ori
H
I
Right
A
D
Intra-RT4
E
Ter
dif
C
B
Left
J
G
F

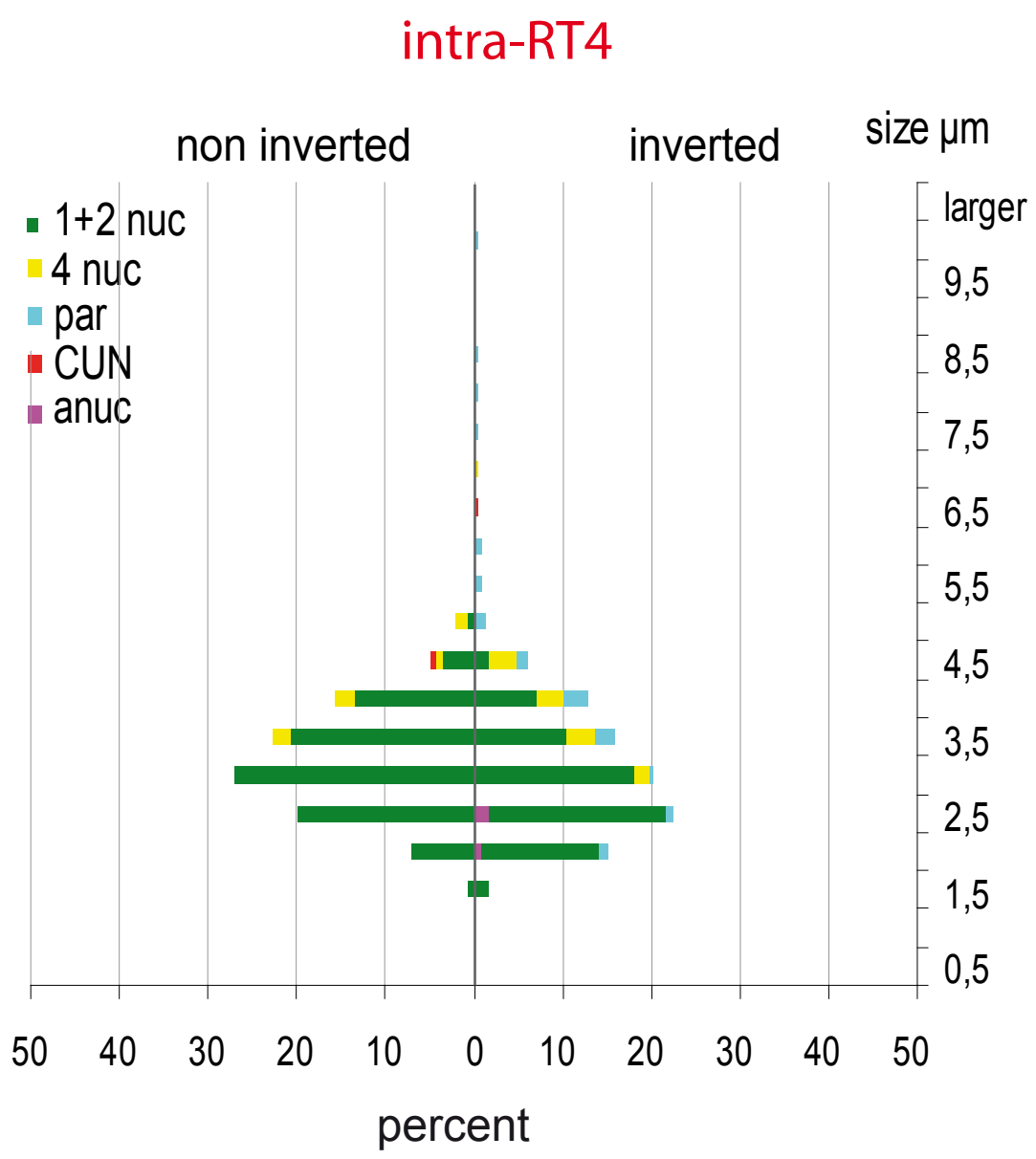
intra-RT4
non inverted
inverted
size µm
1+2 nuc
4 nuc
par
CUN
anuc
larger
9,5
8,5
7,5
6,5
5,5
4,5
3,5
2,5
1,5
0,5
50 40 30 20 10 0 10 20 30 40 50
percent

Figure S6
