# Supplementary material for: Chromosome Structuring Limits Genome Plasticity in Escherichia coli
Source: PLoS Genet. 2007 Dec 14;3(12):e226. doi: 10.1371/journal.pgen.0030226 (PMC2134941; doi:10.1371/journal.pgen.0030226)
Supplement: Supplementary file 9 [file pgen.0030226.st001.pdf]

Supplementary Table 1 (Esnault et al)

Table 1: Strains and plasmids used in this study

Strain (Lab Name)	*att*L/*att*R coordinates in min	Genotype	reference
DY330	----	W3110 Δ*lacU169 gal490* λcI^{857} Δ(*cro-bioA*)	Yu et al., (2000)
FBG140	B17.3	MG1655 Δ*lacIZ* Δ*att*B$^{\lambda}$ ::*aadA*-P_{GBM3}-*lacZ*::*att*B$^{\lambda}$-*cat*	Valens et al., (2004)
FBG146	R17.3	MG1655 Δ*lacIZ* Δ*att*B$^{\lambda}$::*aadA*-*att*R$^{\lambda}$-'*lacZ-cat*	Valens et al., (2004)
FBG146^{off}	R17.3	MG1655 Δ*lacIZ* Δ*att*B$^{\lambda}$::*aadA*-*att*R$^{\lambda}$-'*lacZ-cat*	Valens et al., (2004)
FBG147	R22.7	MG1655 Δ*lacIZ* Δ*att*B$^{\lambda}$::*aadA*-*att*B^{HK022}::*att*R$^{\lambda}$-'*lacZ-cat*	Valens et al., (2004)
FBG150	---	MG1655 Δ*lacIZ* Δ*att*B$^{\lambda}$::*aadA*	Valens et al., (2004)
FBG151	R17.3	MG1655 Δ*lacIZ* Δ*att*B$^{\lambda}$::*aadA*-*att*R$^{\lambda}$-'*lacZ*	This work
FBG152	R17.3	MG1655 Δ*lacIZ* Δ*att*B$^{\lambda}$::*aadA*-*att*R$^{\lambda}$-'*lacZ*	This work
FBG150-*att*L_{7} (LC4)	L7.1	FBG150 (*betT-yahA*)::mini-Tn*5*-*cat*-P_{GBM3}-*lacZ*'-*att*L$^{\lambda}$	Valens et al., (2004)
FBG150-*att*L_{23} (LC13)	L23.6	FBG150*ycdZ*::mini-Tn*5*-*cat*-P_{GBM3}-*lacZ*'-*att*L$^{\lambda}$	This work
FBG150-*att*L_{29} (LC3)	L29.7	FBG150(*ycjV-ompG*)::mini-Tn*5*-*cat*-P_{GBM3}-*lacZ*'-*att*L$^{\lambda}$	Valens et al., (2004)
FBG150-*att*L_{35} (LC19)	L35.1	FBG150*pinQ*::mini-Tn*5*-*cat*-P_{GBM3}-*lacZ*'-*att*L$^{\lambda}$	This work
FBG150-*att*R_{53} (R112)	R53.2	FBG150*cmtA*::mini-Tn*5*-*cat*-P_{GBM3}-*lacZ*'-*att*L$^{\lambda}$	Valens et al., (2004)
FBG150-*att*L_{70} (LC12)	L70.0	FBG150 *yhaI*::mini-Tn*5*-*cat*-P_{GBM3}-*lacZ*'-*att*L$^{\lambda}$	Valens et al., (2004)
FBG150-*att*L_{87} (attL15)	L86.7	FBG150 (*ubiB-fadA*)::mini-Tn10-*cat*-P_{GBM3}-*lacZ*'-*att*L$^{\lambda}$	Valens et al., (2004)
FBG161	L17.3	MG1655 Δ*lacIZ* Δ*att*B$^{\lambda}$ ::*aadA*-P_{GBM3}-*lacZ-att*L$^{\lambda}$	This work
FBG162	L17.3	MG1655 Δ*lacIZ* Δ*att*B$^{\lambda}$ ::*aadA*-P_{GBM3}-*lacZ-att*L$^{\lambda}$	This work
AB1157	----	(F-) *thr-1*, *leuB6*, *thi-1*, Δ(*gpt-proA*)62, *hisG4* (ochre), *argE3*, *mgl-51*, *lacY1*, *galK2*, *ara-14*, *xyl-5*, *mtl-1*, *tsx-33*, *rpsL31*, *supE44*, *kdgK51*, *rfbD1*	Lab collection
649701	----	*recA938*::*cat* miniF-*recA*+	Marsh and Walker (1987)
JJC275	----	AB1157 Δ(*recA-srl*)::Tn*10* mini-F *recA*+, Tc^{R} Amp^{R}	B. Michel
JJC777	----	AB1157 *thr+ pro+ ladR recB268*::Tn*10* pDW52-*recBCD+*	B. Michel
JJC443	----	*mal::Tet lexA Ind-*	B. Michel
JJC2062	----	*recF::Tn5 zid*::miniTn*10*	B. Michel
JJC2280	----	JJC440 *recR*::kan zbb3055::Tn10	B. Michel
JJC2301	----	JJC440 *recO*1504::Tn5 zfh208::Tn10	B. Michel
JJC372	----	AB1157 *recA730 srl::Tn10 lexA def51 mal::cat*, Tc^{R} Cm^{R}	B. Michel
DY330ΔTerA	----	DY330 ΔTerA::*frt-cat-frt*	This work
DY330ΔTerD	----	DY330 ΔTerD::*frt-cat-frt*	This work
DY330ΔTerE	----	DY330 ΔTerE::*frt-cat-frt*	This work
DY330ΔTerH	----	DY330 ΔTerH::*frt-cat-frt*	This work
DY330ΔTerI	----	DY330 ΔTerI::*frt-cat-frt*	This work
DY330 Δ*sfiA*	----	Δ*sfiA*::*frt-cat-frt*	This work
DY330 *tus*	----	DY330 *tus*::*frt-cat-frt*	This work
FBG151 Δ*tus*	----	Δ*tus*::*tetR*	This work
JJC256	----	JJC440 Δ*tus*::*kanR*	Bidnenko et al., (2000)
LN2772	----	Δ*dif*::*tetR*	F. Cornet

Multiple RFT

Intra R1 (FBG151-L161)	R17.3-L26.4	Spc^{R}, Kan^{R}	Valens et al., (2004)
Intra R1 *recA*	R17.3-L26.4	*recA938*::*cat*, Cm^{R}	This work
Intra R1 *recBC*	R17.3-L26.4	*recB268*::Tn*10*, Tc^{R}	This work
Intra R1 *sfiA*	R17.3-L26.4	Δ*sfiA*	This work
Intra R1 *sfiA lexA ind-*	R17.3-L26.4	Δ*sfiA lexA ind- mal::tetR,* Tc^{R}	This work

Intra R1 *recF*	R17.3-L26.4	*recF*::Tn*5* *zid*::Tn*10*, Kn^R Tc^R	This work
Intra R1 *recO*	R17.3-L26.4	*recO1504*::Tn*5* *zfh208*::Tn*10*, Kn^R Tc^R	This work
Intra R1 *recR*	R17.3-L26.4	*recR*::*kan* *zbb3055*::Tn*10*, Kn^R Tc^R	This work
Intra R1 *sfiA recA lexA*def51	R17.3-L26.4	Δ*sfiA* Δ(*recA-srl*)::Tn*10 lexAdef51 mal::cat*, Tc^R Cm^R	This work
Intra R1 ΔTerE	R17.3-L26.4	ΔTerE	This work
Intra R1 *tus*	R17.3-L26.4	Δ*tus*::*tetR*, Tc^R	This work
Intra R2 (FBG152-LC13)	R17.3-L23.6	Spc^R, Cm^R	This work
Intra R2 *recA*	R17.3-L23.6	Δ(*recA-srl*)::Tn*10*, Tc^R	This work
Intra R2 *tus*	R17.3-L23.6	Δ*tus*::*kanR*, Kan^R	This work
Intra R-NS^{right}1 (FBG146-L159)	R17.3-L8.8	Spc^R, Kan^R	Valens et al., (2004)
Intra R-NS^{right}2 (FBG151-LC4)	R17.3-L7.1	Spc^R, Cm^R	This work
Intra R-NS^{right}3 (FBG151-L140)	R17.3-L6.8	Spc^R, Kan^R	This work
Intra R-NS^{right}3 ΔTerH	R17.3-L6.8	ΔTerH, Cm^R	This work
Intra R-NS^{right}3 ΔTerHI	R17.3-L6.8	ΔTerHI, Cm^R	This work
Intra R-NS^{right}3 *recA*	R17.3-L6.8	*recA938*::*cat*, Cm^R	This work
Intra R-NS^{right}3	R17.3-L6.8	Δ*sfiA*	This work
Intra R-NS^{right}3 *tus*	R17.3-L6.8	Δ*tus*::*frt-cat-frt*	This work
Intra R-NS^{right}4 (FBG146-L103)	R17.3-L5.4	Spc^R, Kan^R	Valens et al., (2004)
Intra L-NS^{left}1 (R112-C660)	R53.2-L74.4	Spc^R Kan^R Cm^R	Valens et al., (2004)

Replication arms imbalance

Inter R-L1 (FBG151-L44)	R17.3-L48.3	Spc^R, Kan^R	This work
Inter R-L1 *recA*	R17.3-L48.3	*recA938*::*cat*, Cm^R	This work
Inter R-L1 *sfiA*	R17.3-L48.3	*ΔsfiA*	This work
Inter R-L2 (FBG151-L67)	R17.3-L53.4	Spc^R, Kan^R	This work
Inter R-L2 *recA*	R17.3-L53.4	*recA938*::*cat*, Cm^R	This work
Inter R-L2 *sfiA*	R17.3-L53.4	*ΔsfiA*	This work
Inter NS^{left}-O1(LC12-R11)	L70.0-R1.2	Spc^R, Kan^R, Cm^R	Valens et al., (2004)
Inter NS^{left}-O1*recA*	L70.0-R1.2	Δ(*recA-srl*)::Tn*10*, Tc^R	This work
Inter R-L3 (FBG151-L126)	R17.3-L55.4	Spc^R, Kan^R	This work
Inter R-L3 *recA*	R17.3-L55.4	*recA938*::*cat*, Cm^R	This work
Inter R-L3 *sfiA*	R17.3-L55.4	*ΔsfiA*	This work
Inter R-L4 (FBG151-L54)	R17.3-L45.5	Spc^R, Kan^R	This work
Inter R-L4 *recA*	R17.3-L45.5	*recA938*::*cat*, Cm^R	This work
Inter NSright-NS^{left}1 (LC4-R10)	L7.1-R70.2	Spc^R, Kan^R, Cm^R	Valens et al., (2004)
Inter NSright-NS^{left}1 *recA*	L7.1-R70.2	Δ(*recA-srl*)::Tn*10*, Tc^R	This work
Inter NSright-NS^{left}2 (LC12-R13)	R6.5- L70.0	Spc^R, Kan^R, Cm^R	Valens et al., (2004)
Inter NSright-NS^{left}2 *recA*	R6.5- L70.0	Δ(*recA-srl*)::Tn*10*, Tc^R	This work
Inter R-T1 (FBG151-L1)	R17.3-L38.4	Spc^R, Kan^R	This work
Inter R-T1 *recA*	R17.3-L38.4	*recA938*::*cat*, Cm^R	This work
Inter R-T1 *sfiA*	R17.3-L38.4	*ΔsfiA*	This work
Inter R-L5 ((FBG151-L119)	R17.3-L60.4	Spc^R, Kan^R	This work
Inter R-L5 *recA*	R17.3-L60.4	*recA938*::*cat*, Cm^R	This work
Inter R-T2 (FBG152-LC19)	R17.3-L35.1	Spc^R, Cm^R	This work
Inter R-T2 *recA*	R17.3-L35.1	Δ(*recA-srl*)::Tn*10*, Tc^R	This work
Inter R-T2Δ*dif*	R17.3-L35.1	Δ*dif*, Tc^R	This work
Inter R-T3 (FBG140-SP17)	B17.3-P34.3	Spc^R, Kan^R	Valens et al., (2004)
Inter R-T3 *recA*	B17.3-P34.3	Δ(*recA-srl*)::Tn*10*, Tc^R	This work
Inter R-T4 (FBG146^{off}-R45)	R17.3-L34.2	Spc^R, Kan^R	This work
Inter R-T4 *recA*	R17.3-L34.2	*recA938*::*cat*, Cm^R	This work
Inter L-T1 (LC3-R112)	L29.7-R53.2	Spc^R, Kan^R, Cm^R	Valens et al., (2004)
Inter L-T1 *recA*	L29.7-R53.2	Δ(*recA-srl*)::Tn*10*, Tc^R	This work
Inter R-NS^{left}1 (FBG151-L102)	R17.3-L65.6	Spc^R, Kan^R	This work
Inter R-NS^{left}1 *recA*	R17.3-L65.6	*recA938*::*cat*, Cm^R	This work
Inter R-NS^{left}1 *sfiA*	R17.3-L65.6	*ΔsfiA*	This work
Inter NSright-NS^{left}3 (LC12-R31)	R12.9-70.0	Spc^R, Kan^R, Cm^R	Valens et al., (2004)
Inter NSright-NS^{left}3 *recA*	R12.9-70.0	Δ(*recA-srl*)::Tn*10*, Tc^R	This work
Inter L-T2 (LC3-R123)	L29.7-R58.9	Spc^R, Kan^R, Cm^R	Valens et al., (2004)

Inter L-T2 *recA*	L29.7-R58.9	Δ(*recA-srl*)::Tn*10*, Tc^{R}	This work
Inter R-NS^{left}2 (FBG151-L30)	R17.3-L70.8	Spc^{R}, Kan^{R}	This work
Inter R-NS^{left}2 *recA*	R17.3-L70.8	*recA938*::*cat*, Cm^{R}	This work
Inter R-NS^{left}2 *recBC*	R17.3-L70.8	*recB268*::Tn*10*, Tc^{R}	This work
Inter R-NS^{left}2 *sfiA*	R17.3-L70.8	*ΔsfiA*	This work
Inter R-NS^{left}2 *tus*	R17.3-L70.8	Δ*tus::tetR*, Tc^{R}	This work
Inter R-NS^{left}2 *recA tus*	R17.3-L70.8	Δ*tus*::*tetR recA938*::*cat*, Cm^{R}	This work
Inter T-NS^{right}1 (LC19-R136)	R6.3 –L35.1	Spc^{R}, Kan^{R}, Cm^{R}	This work
Inter T-NS^{right}1 *recA*	R6.3 –L35.1	Δ(*recA-srl*)::Tn*10*, Tc^{R}	This work
Inter T-NS^{left}1(LC3-R137)	L29.7-R64.5	Spc^{R}, Kan^{R}, Cm^{R}	Valens et al., (2004)
Inter T-NS^{left}1 *recA*	L29.7-R64.5	Δ(*recA-srl*)::Tn*10*, Tc^{R}	This work
Inter R-NS^{left}4 (FBG151-L36)	R17.3-L79.0	Spc^{R}, Kan^{R}	This work
Inter R-NS^{left}4 *recA*	R17.3-L79.0	Δ(*recA-srl*)::Tn*10*, Tc^{R}	This work
Inter R-NS^{left}4 *recBC*	R17.3-L79.0	*recB268*::Tn*10*, Tc^{R}	This work
Inter R-NS^{left}4 *tus*	R17.3-L79.0	Δ*tus*::*frt-cat-frt*, Cm^{R}	This work
Inter R-NS^{left}4 *recA tus*	R17.3-L79.0	Δ*tus*::*frt-cat-frt*, Δ(*recA-srl*)::Tn*10*, Cm^{R} Tc^{R}	This work
Inter R-NS^{left}4 *sfiA*	R17.3-L79.0	*ΔsfiA*	This work
Inter R-NS^{left}4 *sfiA recA*	R17.3-L79.0	Δ(*recA-srl*)::Tn*10*, Tc^{R}	This work
Inter R-NS^{left}4 *sfiA recA lex*Adef	R17.3-L79.0	Δ(*recA-srl*)::Tn*10*, *lexAdef51 mal::cat*, Tc^{R} Cam^{R}	This work
Inter R-O1 (FBG151-L120)	R17.3-L82.7	Spc^{R}, Kan^{R}	This work
Inter R-O1 *recA*	R17.3-L82.7	*recA938*::*cat*, Cm^{R}	This work
Inter R-O1 *recBC*	R17.3-L82.7	*recB268*::Tn*10*, Tc^{R}	This work
Inter R-O1 *tus*	R17.3-L82.7	Δ*tus*::*frt-cat-frt*, Cm^{R}	This work
Inter R-O1 *recA tus*	R17.3-L82.7	Δ*tus*::*frt-cat-frt*, Δ(*recA-srl*)::Tn*10*, Cm^{R} Tc^{R}	This work
Inter R-O1 *sfiA*	R17.3-L82.7	*ΔsfiA*	This work
Inter R-O1 *sfiA recA*	R17.3-L82.7	Δ(*recA-srl*)::Tn*10*, Tc^{R}	This work
Inter R-O1 *sfiA recA lexAdef51*	R17.3-L82.7	Δ(*recA-srl*)::Tn*10 lexAdef51 mal::cat*, Tc^{R} Cam^{R}	This work

Inversion inside replichore

Intra O-NS^{right}1 (attL15-R13)	L86.7-R6.5	Spc^{R}, Kan^{R}, Cm^{R}	Valens et al., (2004)
Intra O-NS^{right}1 *recA*	L86.7-R6.5	Δ(*recA-srl*)::Tn*10*, Tc^{R}	This work
Intra O-NS^{right}2 (LC4-R75)	R87.1-L7.1	Spc^{R}, Kan^{R}, Cm^{R}	Valens et al., (2004)
Intra O-NS^{right}2 *recA*	R87.1-L7.1	Δ(*recA-srl*)::Tn*10*, Tc^{R}	This work
Intra O-NS^{right}3 (LC4-R45)	R92.7-L7.1	Spc^{R}, Kan^{R}, Cm^{R}	Valens et al., (2004)
Intra O-NS^{right}3 *recA*	R92.7-L7.1	Δ(*recA-srl*)::Tn*10*, Tc^{R}	This work
Intra L-NS^{left}1 (R112-C660)	R53.2-L74.4	Spc^{R}, Kan^{R}, Cm^{R}	Valens et al., (2004)
Intra L-NS^{left}1*recA*	R53.2-L74.4	Δ(*recA-srl*)::Tn*10*, Tc^{R}	This work
Intra O-NS^{right}4 (attL15-R8)	L87.6-R12.3	Spc^{R}, Kan^{R}, Cm^{R}	Valens et al., (2004)

Ter domain

Intra R-T1 (FBG151-L17)	R17.3-L28.9	Spc^{R}, Kan^{R}	
Intra R-T1 *recA*	R17.3-L28.9	*recA938*::*cat*, Cm^{R}	This work
Intra R-T1 *recBC*	R17.3-L28.9	*recB268*::Tn*10*, Tc^{R}	This work
Intra R-T1 *recF*	R17.3-L28.9	*recF*::Tn*5 zid*::Tn*10*, Kn^{R} Tc^{R}	This work
Intra R-T1 *recO*	R17.3-L28.9	*recO1504*::Tn*5 zfh208*::Tn*10*, Kn^{R} Tc^{R}	This work
Intra R-T1 *recR*	R17.3-L28.9	*recR*::*kan zbb3055*::Tn*10*, Kn^{R} Tc^{R}	This work
Intra R-T1 *sfiA*	R17.3-L28.9	*ΔsfiA*	This work
Intra R-T1 *sfiA lexA ind-*	R17.3-L28.9	*ΔsfiA mal::Tet lexA Ind-*	This work
Intra R-T1 *sfiA recA lexA* def	R17.3-L28.9	Δ*sfiA* Δ(*recA-srl*)::Tn*10 lexAdef51 mal::cat*, Tc^{R} Cm^{R}	This work
Intra R-T1 *tus*	R17.3-L28.9	Δ*tus*::*tetR*, Tc^{R}	This work
Intra R-T1 *recA tus*	R17.3-L28.9	Δ*tus*::*tetR recA938*::*cat*, Tc^{R} Cm^{R}	This work
Intra R-T2 (FBG151-LC3)	R17.3-L29.7	Spc^{R}, Cm^{R}	This work
Intra R-T2 *recA*	R17.3-L29.7	Δ(*recA-srl*)::Tn*10*, Tc^{R}	This work
Intra R-T2 Δ*dif*	R17.3-L29.7	Δ*dif*, Tc^{R}	This work
Intra R-T2 Δ*dif dif*^{RFT}	R17.3-L29.7	Δ*dif dif*^{RFT}, Tc^{R}	This work
Intra R-T3 (FBG151-L160)	R17.3-L31.5	Spc^{R}, Kan^{R}	This work

Intra R-T3 *recA*	R17.3-L31.5	*recA938*::*cat,* Cm^{R}	This work
Intra T1 (LC3-R58)	R26.0-L29.7	Spc^{R}, Kan^{R}, Cm^{R}	This work
Intra T2 (LC3-R177)	R26.4-L29.7	Spc^{R}, Kan^{R}, Cm^{R}	This work
Intra R3 (LC13-R26 ΔTerAD)	R19.0-L23.6	ΔTerA ΔTerD, Spc^{R}, Kan^{R}, Cm^{R}	This work
Intra R3 *recA*	R19.0-L23.6	Δ(*recA-srl*)::Tn*10,* Tc^{R}	This work
Intra R4 (LC13-R64 ΔTerAD)	R21.7-L23.6	ΔTerA ΔTerD, Spc^{R}, Kan^{R}, Cm^{R}	This work
Intra R4 *recA*	R21.7-L23.6	Δ(*recA-srl*)::Tn*10,* Tc^{R}	This work
Intra R-T6 (FBG147-L17)	R22.7-L28.9	Spc^{R}, Apra^{R}, Kan^{R}	Valens et al., (2004)
Intra R-T6 *recA*	R22.7-L28.9	Δ(*recA-srl*)::Tn*10,* Tc^{R}	This work

Ori domain

Intra-OR1 (FBG151-L11)	L0.7-R17.3	Spc^{R}, Kan^{R}	This work
Intra-OR1 *recA*	L0.7-R17.3	*recA938*::*cat,* Cm^{R}	This work
Intra-OR1 *recBC*	L0.7-R17.3	*recB268*::Tn*10*, Tc^{R}	This work
Intra-OR1 *sfiA*	L0.7-R17.3	*ΔsfiA*	This work
Intra-OR1ΔterH	L0.7-R17.3	ΔterH	This work
Intra-OR1 ΔterHI	L0.7-R17.3	ΔterHI	This work
Intra-OR2 (FBG151-L28)	L97.0-R17.3	Spc^{R}, Kan^{R}	This work
Intra-OR2 *recA*	L97.0-R17.3	*recA938*::*cat,* Cm^{R}	This work
Intra-OR2 *recBC*	L97.0-R17.3	*recB268*::Tn*10*, Tc^{R}	This work
Intra-OR2 *sfiA*	L97.0-R17.3	*ΔsfiA*	This work
Intra-OR2 ΔTerH	L97.0-R17.3	ΔterH	This work
Intra-OR2 ΔTerH *recA*	L97.0-R17.3	ΔterH Δ(*recA-srl*)::Tn*10,* Tc^{R}	This work
Intra-OR2 ΔTerHI	L97.0-R17.3	ΔterHI	This work
Intra-OR2 ΔTerHI *recA*	L97.0-R17.3	ΔterHI Δ(*recA-srl*)::Tn*10,* Tc^{R}	This work
Intra-OR3 (FBG162-R128)	R95.0-L17.3	Spc^{R}, Kan^{R}	This work
Intra-OR3 *recA*	R95.0-L17.3	Δ(*recA-srl*)::Tn*10,* Tc^{R}	This work
Intra-OR3 *recBC*	R95.0-L17.3	*recB268*::Tn*10*, Tc^{R}	This work
Intra-OR3 ΔTerH	R95.0-L17.3	ΔTerH	This work
Intra-OR3 ΔTerH *recA*	R95.0-L17.3	ΔTerH Δ(*recA-srl*)::Tn*10,* Tc^{R}	This work
Intra-OR3 ΔTerHI	R95.0-L17.3	ΔTerHI	This work
Intra-OR3 ΔTerHI *recA*	R95.0-L17.3	ΔTerHI Δ(*recA-srl*)::Tn*10,* Tc^{R}	This work
Intra-OR4 (FBG161-R32)	R94.2-L17.3	Spc^{R}, Kan^{R}	This work
Intra-OR5 ((FBG161-R45)	R92.7-L17.3	Spc^{R}, Kan^{R}	This work
Intra-OR5 *recA*	R94.2-L17.3	*recA938*::*cat,* Cm^{R}	This work
Intra-OR5 *recBC*	R94.2-L17.3	*recB268*::Tn*10*, Tc^{R}	This work
Intra-OR5 *tus*	R94.2-L17.3	Δ*tus*::*frt-cat-frt*	This work
Intra-OR6 (FBG162-R6)	R88.4-L17.3	Spc^{R}, Kan^{R}	This work
Intra-OR6 ΔTerH	R88.4-L17.3	ΔTerH	This work
Intra-OR6 ΔTerHI	R88.4-L17.3	ΔTerHI	This work
Intra-OR7 (FBG161-R75)	R87.1-L17.3	Spc^{R}, Kan^{R}	This work
Intra-OR7 ΔTerH	R87.1-L17.3	ΔTerH	This work
Intra-OR7 ΔTerHI	R87.1-L17.3	ΔTerHI	This work
Intra-OR8 (attL15-R26)	L86.7-R19.0	Spc^{R}, Cm^{R}, Kan^{R}	This work

Plasmids

PTSA29-CXI-AK	pTSA29 carrying cI^{857}-P$_{R}$-*int*$^{\lambda}$	Valens et al., 2004
pTSA29-CXI	pTSA29 carrying cI^{857}-P$_{R}$-(*xis*$^{\lambda}$-*int*$^{\lambda}$)	Valens et al., 2004
pTSC29-CXI	pTSC29 carrying cI^{857}-P$_{R}$-(*xis*$^{\lambda}$-*int*$^{\lambda}$)	This work
pKO3	pSC101 ts replicon carrying *sacB*, Cm^{R}	Link et al, 1997
pZA-P$_{sfiA}$-*gfp*	P15A replicon carrying P$_{sfiA}$-*gfp*, Cm^{R}	This work
pBAD18-P$_{sfiA}$-*uidA*	pBAD18 carrying P$_{sfiA}$-*uidA*, Amp^{R}	This work
pTSC29-CXI	pTSC29 carrying cI^{857}-P$_{R}$-(*xis*$^{\lambda}$-*int*$^{\lambda}$)	This work
pCP20	pSC101 ts replicon carrying FLP, Amp^{R} Cm^{R}	Cherapenov and Wakernagel (1995)
pKD3	template plasmid carrying *frt-cat-frt*	Datsenko and Wanner (2000)
